# Supplementary material for: Virtual Screening with Gnina 1.0
Source: Molecules. 2021 Dec 4;26(23):7369. doi: 10.3390/molecules26237369 (PMC8659095; doi:10.3390/molecules26237369)
Supplement: Supplementary file 1 [file molecules-26-07369-s001.zip › molecules-1463113-supplementary.pdf]

Supporting Information: Virtual Screening with Gnina 1.0

Jocelyn Sunseri and David R. Koes*

Department of Computational and Systems Biology, University of Pittsburgh, Pittsburgh, PA 15260

E-mail: dkoes@pitt.edu

Per-Target Confidence Intervals

In addition to evaluating the overall performance of the method (Table S2) it is also insightful to look at per-target performance. There has been some debate about how to statistically assess claims made about machine learning model performance for drug discovery tasks like virtual screening.[2,3] The norm has been, as in much of the other recent machine learning literature that reports model performance on community benchmarks, to avoid statistically evaluating them at all. Our approach is to use bootstrapping to compute 95% confidence intervals of the various metrics and the non-parametric Mann-Whitney U test to evaluate if the bootstrapped distributions of metrics differ significantly. For each target, we perform 1000 rounds of bootstrapping using stratified sampling with replacement to address the class imbalance issue. The resulting distributions are used to calculate the 5%-95% confidence interval and are compared with Mann-Whitney U to get a p-value. Confidence intervals are illustrated in Figures S1 – S10.

Table S1: Analysis of LIT-PCBA assays.

Target	Primary PubChem Assay ID	Confirmatory PubChem Assay ID	Publication	Notes
ADRB2	492947	588463	—	**Cell based assay**
ALDH1	1030	493210	—	
ESR1_ago	743075	743077	?	**Cell based assay**
ESR1_ant	743080	743091	?	**Cell based assay**
FEN1	588795	488816	?	Paper describes assay, not results.
GBA	2101	2590	?	Both antagonists and agonists
IDH1	602179	624002	?	Most compounds selective for R132H, not wild type
KAT2A	504327	588347	—	
MAPK1	995	1742	?	**Cell-based assay**. At least one active inhibits EGFR, not MAPK1
MTORC1	493208	651784, 651789, 651791, 651793	?	**Cell based assay**. Paper describes assay, not results
OPRK1	1777	2133,2136	?	**Cell based assay**. Both antagonists and agonists
PKM2	1631	1751	?	Both antagonists and agonists
PPARG	743094	743140	—	**Cell based assay**
TP53	651631	720552	?	**Cell based assay**
VDR	602199	602200, 602201, 602202, 602204	?	Actives reported in paper do not match confirmatory screen compounds.

Table S2: Mean AUCs, NEF1% and EF1% values on DUD-E and LIT-PCBA. The best CNN model value for each column is shown in bold. Models whose distributions of per-benchmark metrics are not statistically dissimilar to the model in bold (as computed with a Mann-Whitney U rank test, p-value > 0.05) are shown in italic. Note that RFScore-VS was trained on DUD-E.

Model	DUD-E			LIT-PCBA		
	AUC	NEF1%	EF1%	AUC	NEF1%	EF1%
RFScore-4	0.678	0.0911	5.5	*0.571*	*0.0224*	*1.67*
RFScore-VS	0.944	0.798	49.3	*0.553*	0.0214	1.75
Vina	0.725	0.167	9.93	*0.57*	*0.0247*	*1.71*
Vinardo	0.748	0.204	12.4	*0.58*	0.0235	1.7
General (Pose)	0.712	0.208	12.9	0.509	*0.0251*	*2.01*
General (Affinity)	0.749	0.229	13.9	*0.594*	*0.0445*	*3.7*
Dense (Pose)	*0.767*	**0.34**	**20.9**	0.527	*0.0415*	*3.45*
Dense (Affinity)	**0.793**	*0.303*	*18.8*	**0.616**	*0.0553*	*4.45*
Default (Pose)	0.753	*0.294*	*18.1*	0.518	*0.0292*	*2.41*
Default (Affinity)	*0.781*	*0.29*	*17.7*	*0.61*	**0.0561**	**4.64**

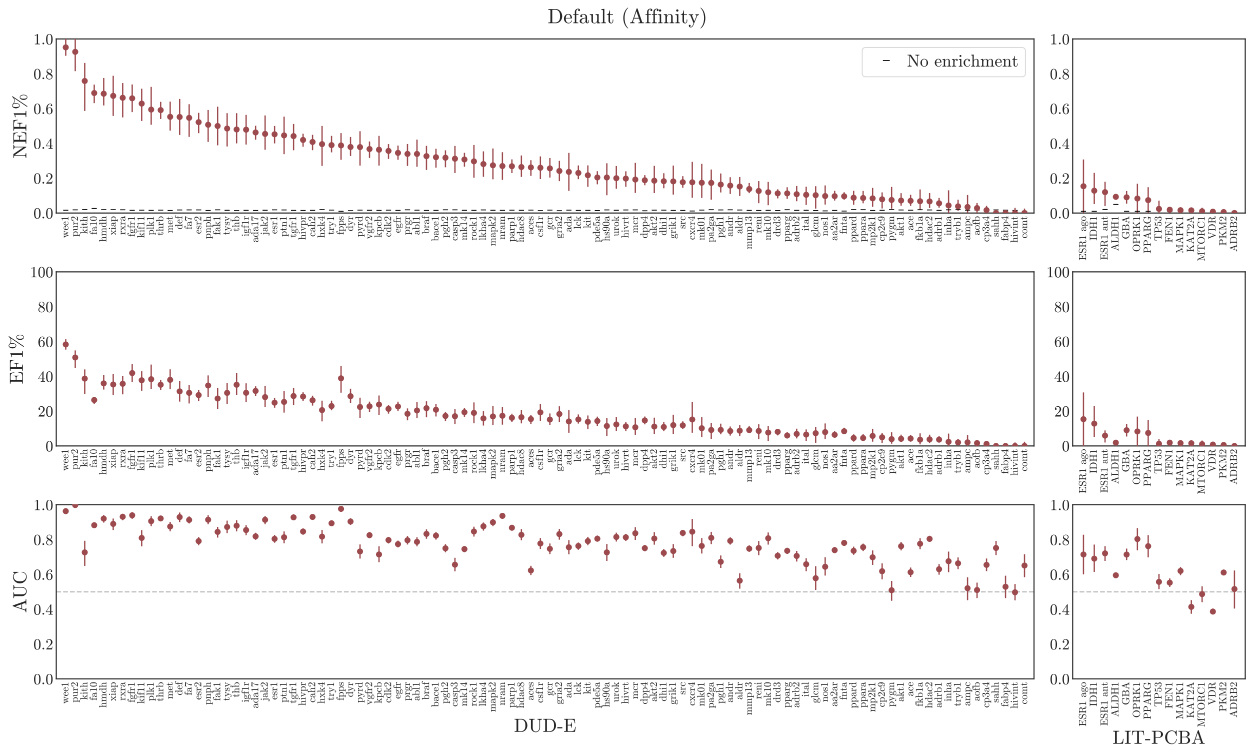

Figure S1: Per-target 5%-95% confidence intervals for various metrics for Default (Affinity).

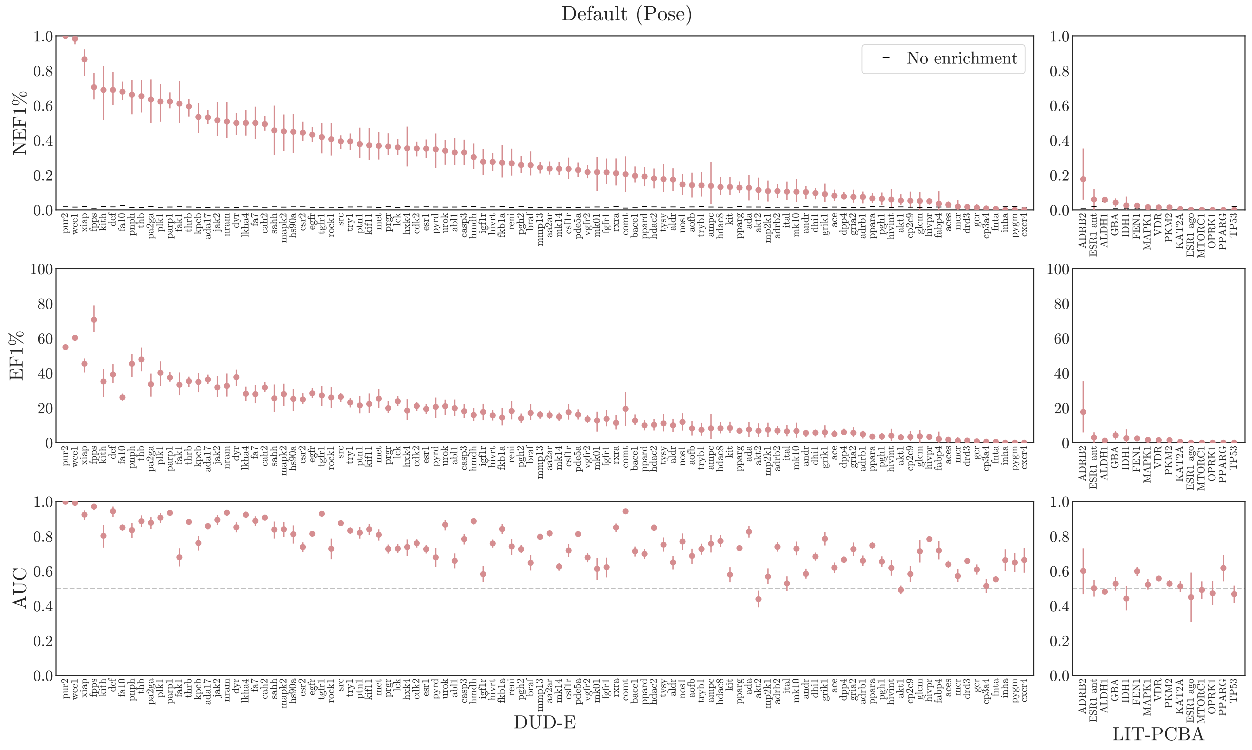

Figure S2: Per-target 5%-95% confidence intervals for various metrics for Default (Pose).

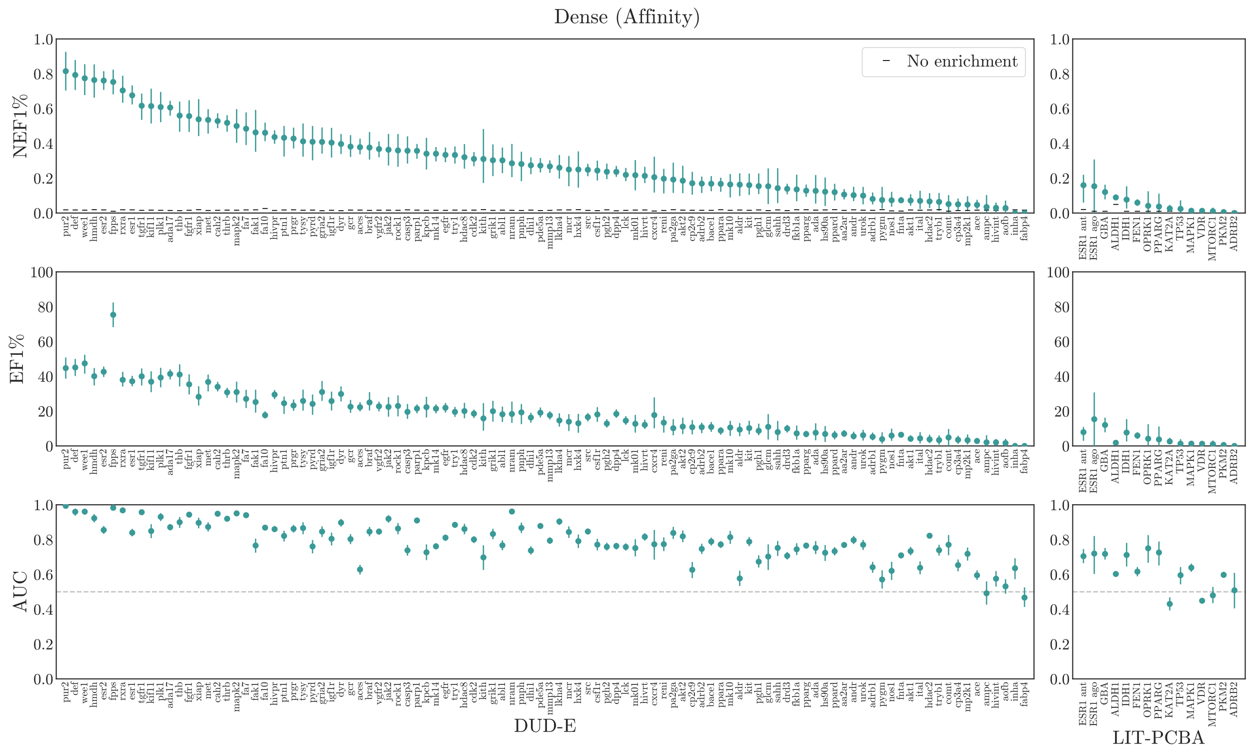

Figure S3: Per-target 5%-95% confidence intervals for various metrics for Dense (Affinity).

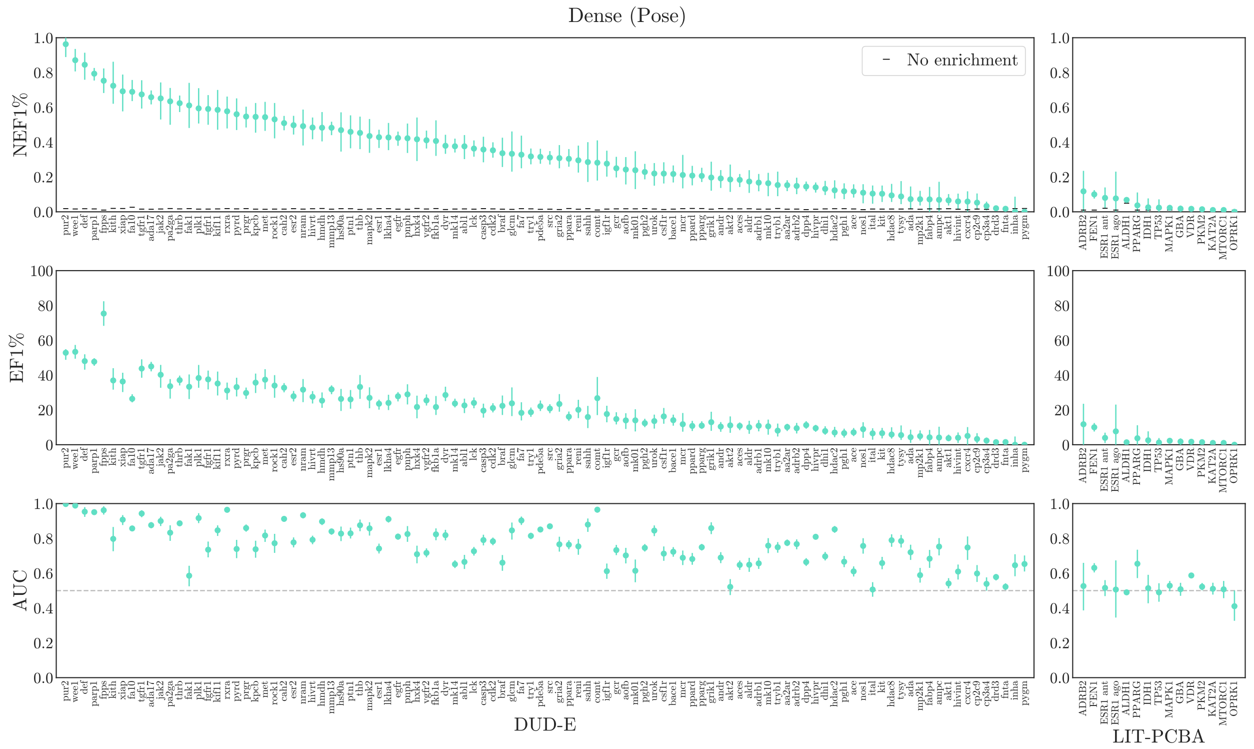

Figure S4: Per-target 5%-95% confidence intervals for various metrics for Dense (Pose).

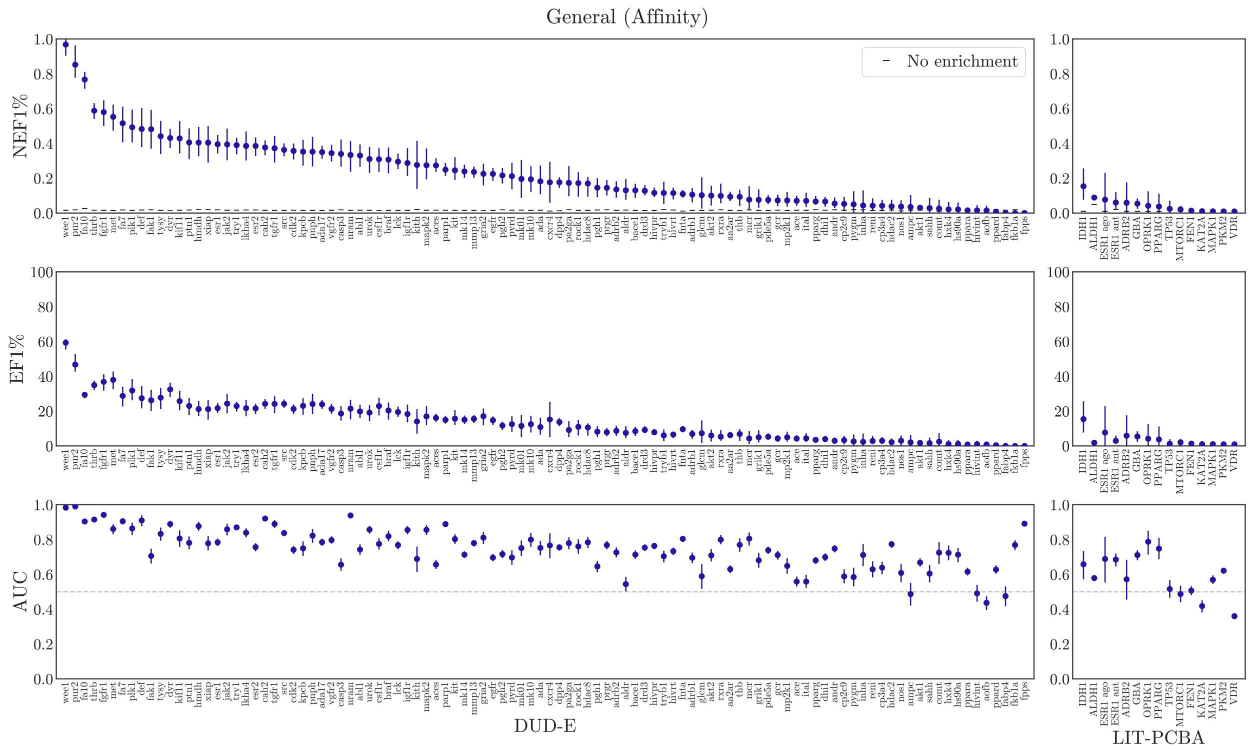

Figure S5: Per-target 5%-95% confidence intervals for various metrics for General (Affinity).

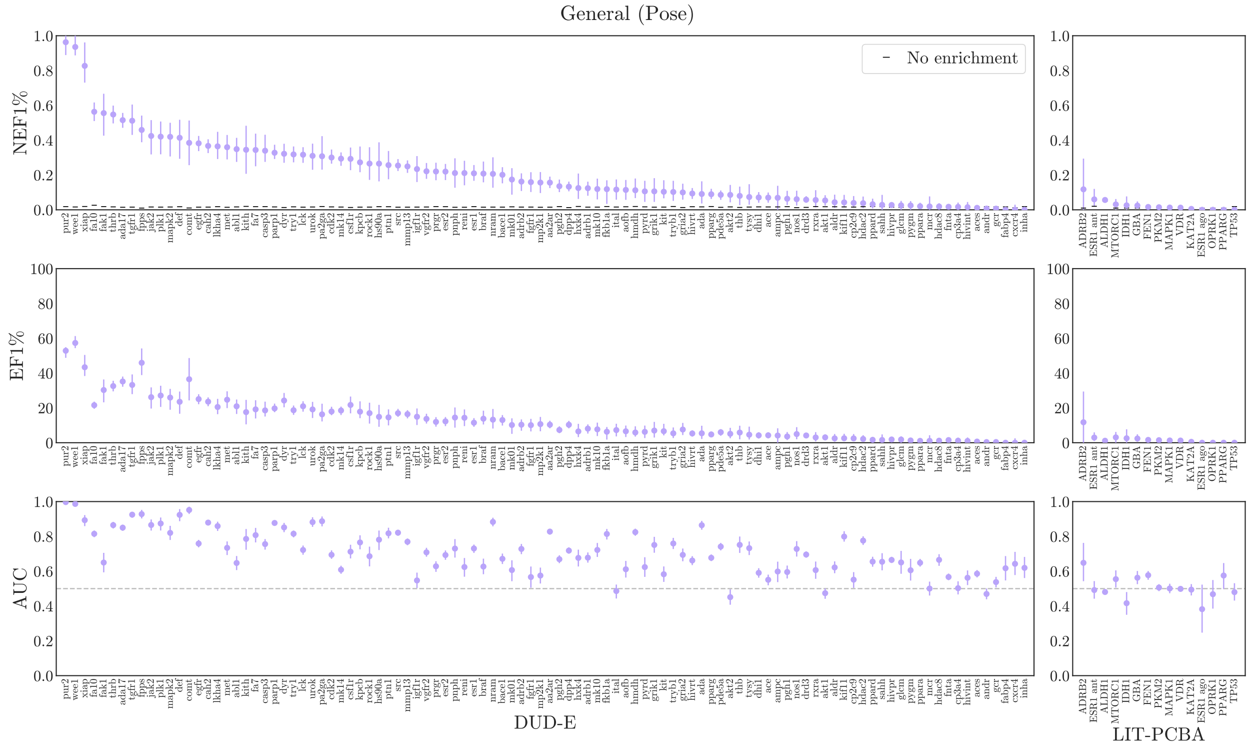

Figure S6: Per-target 5%-95% confidence intervals for various metrics for General (Pose).

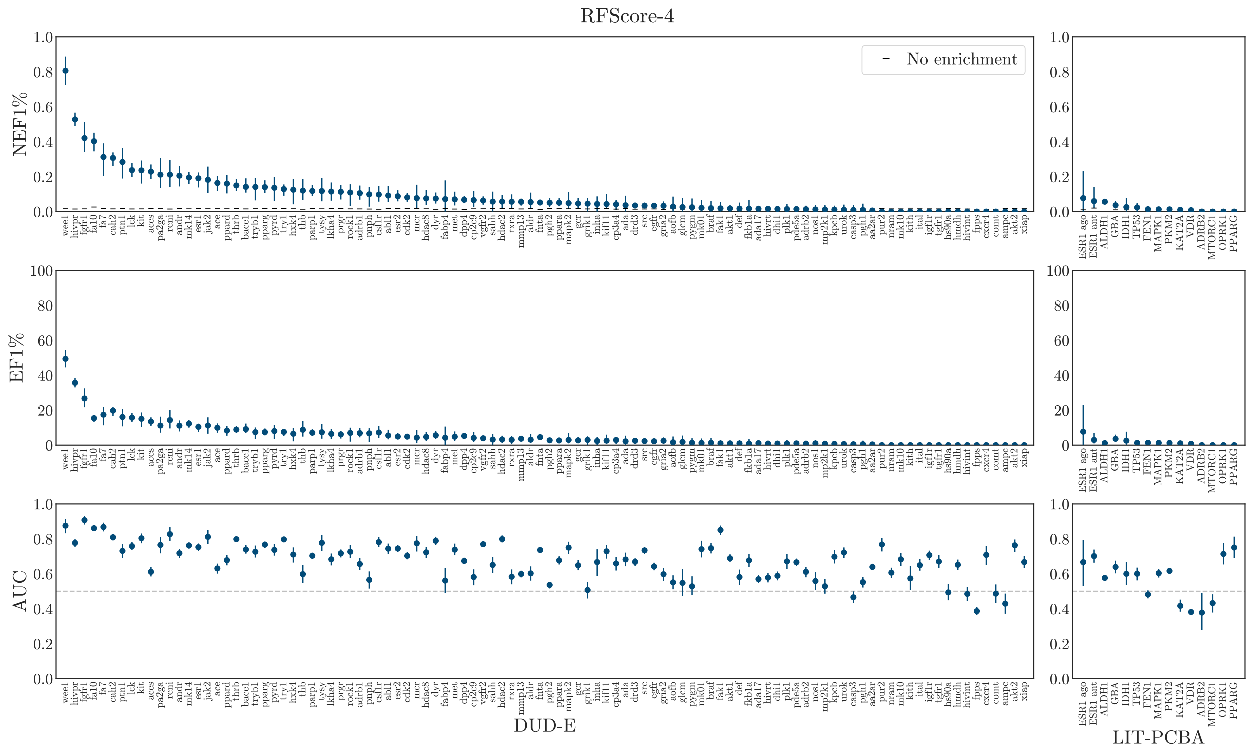

Figure S7: Per-target 5%-95% confidence intervals for various metrics for RFScore-4.

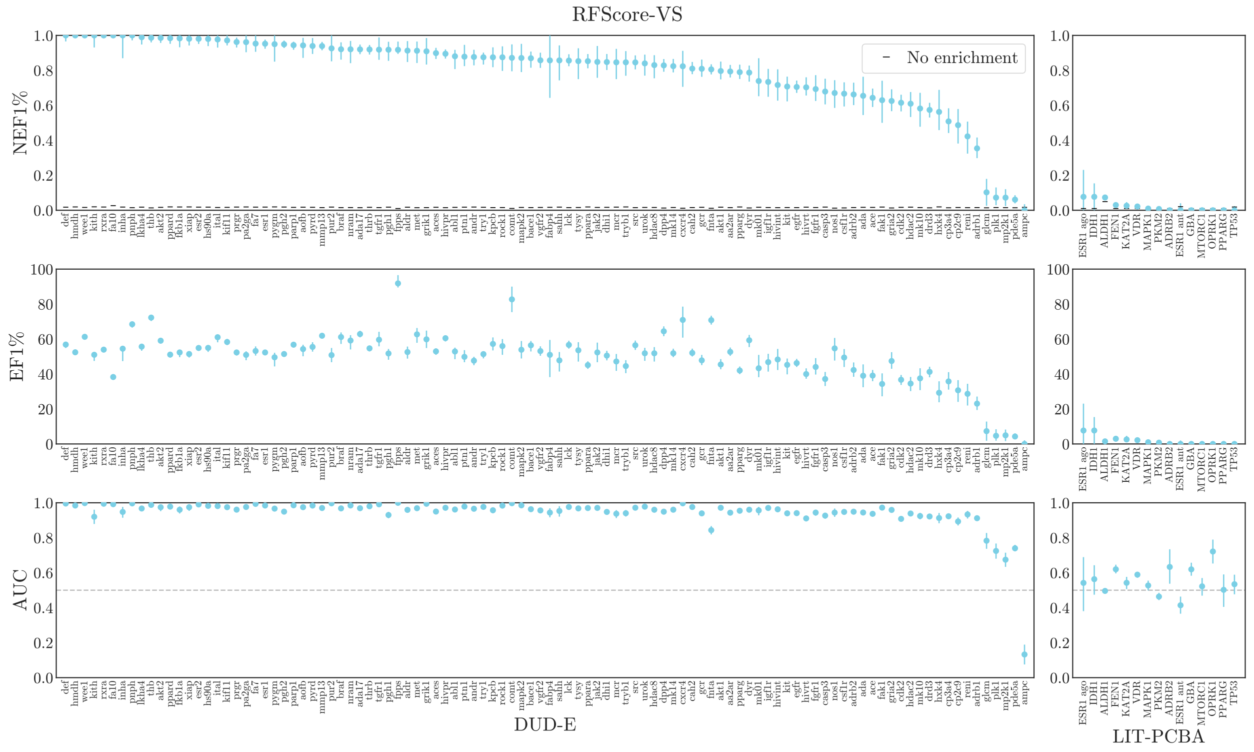

Figure S8: Per-target 5%-95% confidence intervals for various metrics for RFScore-VS.

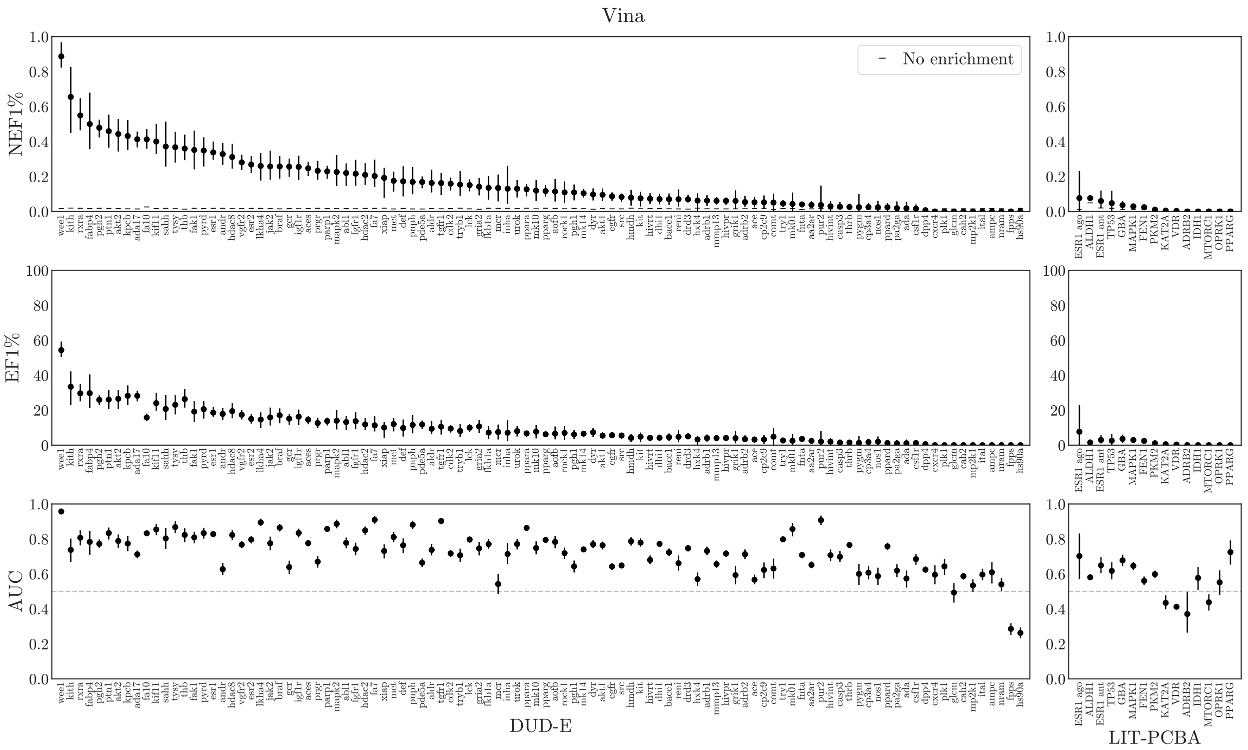

Figure S9: Per-target 5%-95% confidence intervals for various metrics for Vina.

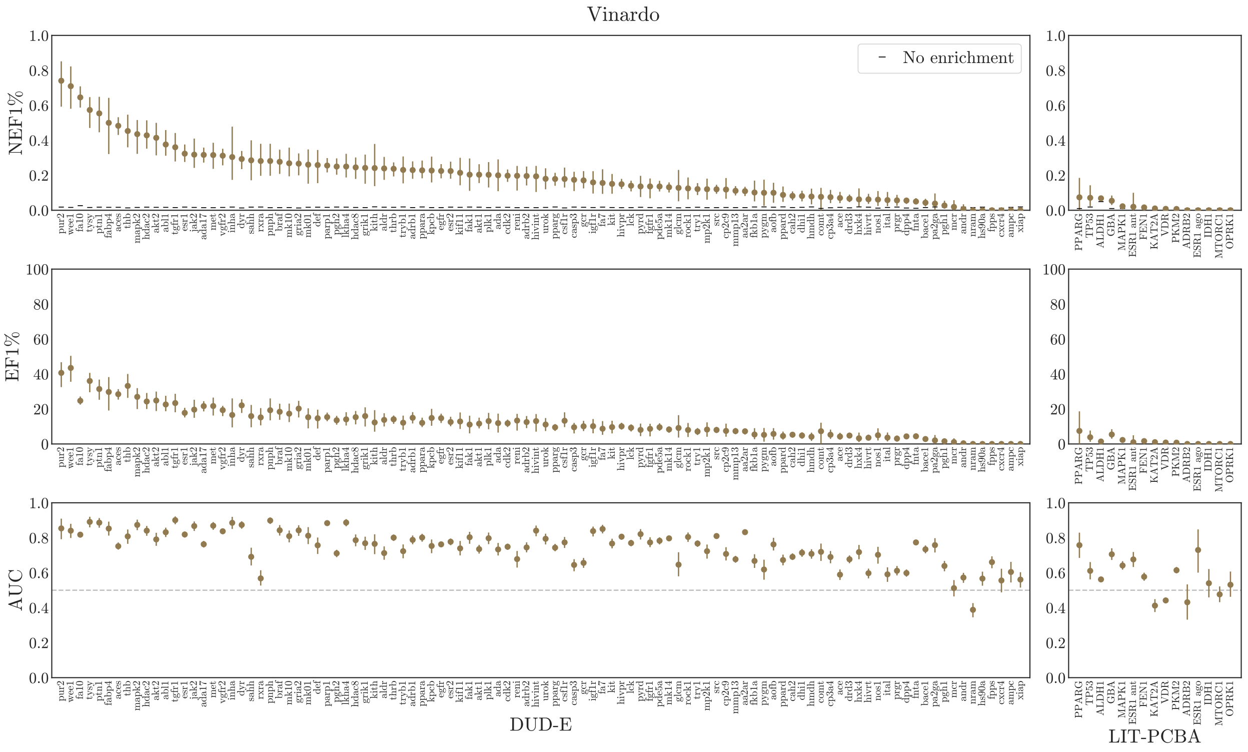

Figure S10: Per-target 5%-95% confidence intervals for various metrics for Vinardo.

NEF1%

	Default (Affinity)	Default (Pose)	Dense (Affinity)	Dense (Pose)	General (Affinity)	General (Pose)	Vina	Vinardo	RFScore-VS	RFScore-4
aa2ar	.072 - .097 - .13	.2 - .24 - .28	.078 - .11 - .14	.12 - .15 - .18	.069 - .094 - .12	.12 - .16 - .19	.019 - .037 - .059	.081 - .11 - .13	.76 - .79 - .83	0 - .0063 - .013
abl1	.27 - .34 - .42	.26 - .33 - .41	.23 - .3 - .38	.3 - .38 - .44	.27 - .33 - .39	.28 - .35 - .41	.15 - .22 - .28	.31 - .38 - .46	.81 - .88 - .92	.055 - .092 - .15
ace	.041 - .07 - .11	.052 - .082 - .12	.023 - .047 - .076	.082 - .12 - .16	.041 - .07 - .099	.041 - .07 - .099	.023 - .053 - .082	.041 - .07 - .11	.59 - .64 - .7	.12 - .16 - .2
aces	.21 - .26 - .3	.015 - .03 - .049	.34 - .38 - .43	.14 - .18 - .22	.24 - .27 - .31	0 - .011 - .022	.2 - .25 - .28	.43 - .48 - .53	.87 - .9 - .93	.19 - .23 - .27
ada	.13 - .24 - .35	.055 - .13 - .2	.055 - .13 - .22	.018 - .073 - .15	.11 - .18 - .27	.036 - .091 - .16	0 - .018 - .055	.11 - .2 - .29	.54 - .65 - .76	0 - .036 - .091
ada17	.42 - .46 - .5	.49 - .53 - .57	.56 - .61 - .64	.62 - .66 - .7	.31 - .35 - .39	.47 - .52 - .56	.37 - .41 - .46	.27 - .32 - .36	.89 - .92 - .95	.0055 - .017 - .028
adrb1	.031 - .056 - .087	.037 - .075 - .11	.05 - .081 - .12	.12 - .17 - .22	.062 - .11 - .14	.081 - .12 - .16	.031 - .062 - .093	.18 - .23 - .28	.3 - .35 - .42	.068 - .11 - .15
adrb2	.068 - .11 - .16	.068 - .11 - .15	.11 - .17 - .21	.1 - .15 - .2	.088 - .14 - .18	.11 - .16 - .21	.027 - .054 - .088	.14 - .2 - .25	.6 - .66 - .73	0 - .014 - .034
akt1	.042 - .072 - .11	.03 - .054 - .09	.042 - .072 - .11	.036 - .066 - .1	.012 - .03 - .048	.024 - .054 - .084	.06 - .096 - .13	.16 - .2 - .26	.75 - .8 - .85	0 - .018 - .036
akt2	.11 - .19 - .27	.057 - .11 - .19	.11 - .19 - .27	.11 - .19 - .27	.043 - .1 - .16	.043 - .086 - .14	.34 - .44 - .53	.31 - .41 - .5	.96 - .99 - 1	0 - 0 - 0
aldr	.098 - .15 - .21	.11 - .17 - .24	.098 - .16 - .23	.11 - .17 - .24	.076 - .13 - .2	.011 - .043 - .087	.11 - .16 - .24	.17 - .24 - .3	.85 - .91 - .97	.022 - .054 - .11
ampc	0 - .034 - .1	.034 - .14 - .28	0 - .034 - .1	0 - .069 - .17	0 - .034 - .1	0 - .069 - .14	0 - 0 - 0	0 - 0 - 0	0 - 0 - .034	0 - 0 - 0
andr	.11 - .16 - .21	.062 - .1 - .14	.068 - .1 - .15	.14 - .19 - .24	.027 - .055 - .089	0 - .0068 - .021	.27 - .33 - .39	0 - .0068 - .034	.83 - .88 - .92	.14 - .21 - .26
aofb	0 - .029 - .057	.071 - .14 - .21	0 - .029 - .071	.16 - .24 - .31	0 - .014 - .043	.057 - .11 - .17	.057 - .11 - .19	.043 - .1 - .16	.87 - .94 - .99	0 - .029 - .086
bace1	.26 - .32 - .37	.16 - .2 - .25	.12 - .17 - .21	.17 - .22 - .27	.087 - .13 - .17	.15 - .2 - .24	.038 - .071 - .1	.022 - .043 - .065	.82 - .87 - .91	.11 - .14 - .19
braf	.25 - .33 - .39	.2 - .26 - .34	.31 - .38 - .47	.26 - .34 - .43	.23 - .31 - .38	.16 - .21 - .28	.19 - .26 - .32	.21 - .28 - .35	.86 - .92 - .96	0 - .02 - .059
cah2	.36 - .41 - .45	.46 - .49 - .54	.49 - .53 - .57	.47 - .51 - .55	.33 - .38 - .42	.33 - .37 - .41	0 - 0 - .0032	.057 - .082 - .11	.78 - .81 - .85	.26 - .31 - .34
casp3	.23 - .31 - .39	.26 - .33 - .4	.28 - .36 - .44	.28 - .36 - .43	.27 - .34 - .42	.28 - .34 - .43	.0092 - .028 - .055	.11 - .17 - .22	.61 - .68 - .75	0 - .0092 - .037
cdk2	.31 - .36 - .4	.31 - .35 - .39	.27 - .31 - .35	.31 - .35 - .4	.31 - .36 - .4	.27 - .3 - .35	.12 - .16 - .19	.16 - .2 - .23	.57 - .61 - .66	.057 - .081 - .11
comt	0 - 0 - .026	.1 - .21 - .31	0 - .051 - .1	.18 - .28 - .41	0 - .026 - .077	.26 - .38 - .51	0 - .051 - .1	0 - .077 - .13	.79 - .87 - .95	0 - 0 - 0
cp2c9	.026 - .079 - .12	.013 - .053 - .11	.11 - .17 - .24	.013 - .053 - .11	.013 - .053 - .093	.013 - .039 - .079	.013 - .053 - .092	.066 - .12 - .18	.38 - .49 - .58	.026 - .066 - .12
cp3a4	0 - .017 - .042	0 - .0083 - .025	.017 - .05 - .083	.0083 - .033 - .058	.017 - .042 - .075	0 - .017 - .042	.0083 - .025 - .05	.041 - .075 - .12	.44 - .51 - .58	.0083 - .042 - .067
csf1r	.2 - .26 - .33	.18 - .24 - .3	.19 - .24 - .3	.16 - .22 - .28	.24 - .31 - .37	.23 - .29 - .36	0 - .016 - .041	.13 - .18 - .24	.59 - .67 - .73	.057 - .098 - .15
cxcr4	.088 - .18 - .29	0 - 0 - 0	.12 - .21 - .32	0 - .059 - .12	.059 - .18 - .29	0 - 0 - .029	0 - 0 - 0	0 - 0 - 0	.71 - .82 - .91	0 - 0 - 0
def	.45 - .55 - .66	.6 - .69 - .79	.71 - .79 - .88	.76 - .84 - .91	.38 - .48 - .6	.29 - .41 - .52	.086 - .17 - .26	.16 - .26 - .34	.97 - 1 - 1	0 - .017 - .052
dhi1	.14 - .18 - .23	.061 - .096 - .13	.22 - .27 - .32	.096 - .13 - .18	.036 - .066 - .091	.041 - .071 - .1	.041 - .071 - .1	.051 - .081 - .11	.8 - .85 - .89	.0051 - .015 - .03
dpp4	.16 - .19 - .22	.06 - .077 - .1	.21 - .24 - .27	.12 - .14 - .17	.14 - .18 - .21	.11 - .13 - .16	.0024 - .0072 - .017	.036 - .056 - .072	.79 - .83 - .86	.051 - .068 - .089
drd3	.084 - .11 - .14	.0058 - .017 - .029	.11 - .14 - .17	.0087 - .02 - .032	.1 - .13 - .16	.04 - .058 - .078	.052 - .07 - .096	.046 - .067 - .09	.53 - .57 - .61	.02 - .035 - .049
dyr	.33 - .38 - .44	.43 - .5 - .56	.34 - .4 - .45	.33 - .38 - .44	.37 - .43 - .48	.27 - .32 - .38	.063 - .098 - .13	.24 - .29 - .34	.74 - .79 - .83	.046 - .075 - .11
egfr	.31 - .35 - .39	.39 - .43 - .48	.29 - .33 - .38	.38 - .42 - .46	.19 - .22 - .26	.34 - .38 - .42	.062 - .087 - .11	.18 - .22 - .26	.67 - .71 - .74	.02 - .034 - .051
esr1	.4 - .45 - .5	.3 - .35 - .4	.62 - .68 - .73	.37 - .43 - .47	.34 - .4 - .45	.17 - .21 - .26	.3 - .34 - .4	.28 - .32 - .38	.92 - .95 - .98	.14 - .19 - .22
esr2	.46 - .52 - .58	.4 - .44 - .51	.71 - .76 - .81	.44 - .5 - .55	.33 - .39 - .43	.17 - .22 - .26	.22 - .27 - .32	.18 - .22 - .28	.95 - .98 - 1	.059 - .088 - .12
fa10	.63 - .69 - .74	.63 - .68 - .74	.41 - .46 - .52	.64 - .69 - .76	.71 - .77 - .81	.51 - .56 - .62	.36 - .41 - .47	.59 - .65 - .71	1 - 1 - 1	.34 - .4 - .45
fa7	.44 - .55 - .62	.41 - .5 - .59	.39 - .48 - .58	.25 - .33 - .44	.41 - .52 - .61	.25 - .34 - .44	.14 - .2 - .3	.094 - .16 - .23	.91 - .95 - 1	.2 - .31 - .39
fabp4	0 - 0 - 0	0 - .036 - .11	0 - 0 - 0	0 - .071 - .14	0 - 0 - 0	0 - 0 - 0	.36 - .5 - .68	.32 - .5 - .64	.64 - .86 - 1	0 - .071 - .18
fak1	.39 - .5 - .61	.5 - .61 - .74	.35 - .46 - .59	.48 - .61 - .74	.37 - .48 - .59	.43 - .56 - .67	.24 - .35 - .46	.11 - .2 - .3	.5 - .63 - .74	0 - .019 - .056
fgfr1	.58 - .66 - .74	.15 - .22 - .3	.47 - .56 - .65	.5 - .59 - .67	.5 - .58 - .65	.1 - .16 - .22	.15 - .22 - .3	.068 - .14 - .18	.62 - .69 - .77	.34 - .42 - .51
fkb1a	.017 - .068 - .14	.19 - .27 - .37	.068 - .14 - .22	.32 - .41 - .53	0 - 0 - .017	.034 - .12 - .17	.068 - .14 - .2	.051 - .1 - .17	.93 - .98 - 1	0 - .017 - .068
fnta	.077 - .096 - .12	0 - .0058 - .012	.058 - .073 - .092	.0077 - .017 - .027	.088 - .11 - .13	.0077 - .017 - .027	.025 - .04 - .054	.037 - .05 - .069	.78 - .81 - .83	.037 - .052 - .067
fpps	.31 - .39 - .46	.64 - .71 - .79	.68 - .75 - .82	.68 - .75 - .82	0 - 0 - 0	.39 - .46 - .54	0 - 0 - 0	0 - 0 - 0	.89 - .92 - .96	0 - 0 - 0
gcr	.2 - .26 - .32	0 - .013 - .033	.32 - .38 - .45	.2 - .25 - .32	.039 - .072 - .11	0 - .0066 - .02	.2 - .26 - .3	.12 - .17 - .22	.76 - .81 - .86	.02 - .046 - .079
glcm	.026 - .1 - .15	0 - .051 - .1	.051 - .15 - .26	.23 - .33 - .46	.026 - .1 - .21	0 - .026 - .051	0 - 0 - 0	.051 - .13 - .23	.026 - .1 - .18	0 - .026 - .077
gria2	.18 - .24 - .3	.042 - .075 - .12	.34 - .41 - .49	.25 - .31 - .38	.16 - .23 - .28	.058 - .1 - .15	.092 - .14 - .19	.2 - .27 - .33	.57 - .62 - .69	.0083 - .033 - .058
grik1	.11 - .18 - .27	.045 - .091 - .15	.21 - .3 - .39	.12 - .2 - .29	.03 - .076 - .14	.045 - .11 - .18	.015 - .061 - .12	.15 - .24 - .32	.83 - .91 - .98	0 - .045 - .077
hdac2	.029 - .067 - .11	.12 - .18 - .24	.029 - .067 - .11	.076 - .12 - .18	.0095 - .038 - .076	.0095 - .038 - .076	.15 - .21 - .28	.35 - .43 - .51	.53 - .61 - .68	.019 - .057 - .095
hdac8	.21 - .26 - .33	.085 - .13 - .19	.25 - .32 - .4	.057 - .094 - .16	.094 - .17 - .21	0 - .019 - .038	.25 - .31 - .39	.18 - .25 - .3	.75 - .83 - .89	.038 - .075 - .12
hivint	0 - 0 - .03	.015 - .06 - .12	0 - .03 - .06	.015 - .06 - .1	0 - .015 - .045	0 - .015 - .045	0 - .03 - .06	.1 - .19 - .25	.63 - .72 - .81	0 - 0 - 0
hivpr	.38 - .42 - .46	.03 - .05 - .066	.4 - .44 - .48	.11 - .14 - .17	.091 - .12 - .15	.014 - .028 - .044	.039 - .061 - .08	.12 - .15 - .18	.87 - .9 - .92	.49 - .53 - .57
hivrt	.15 - .2 - .24	.22 - .28 - .33	.17 - .21 - .27	.42 - .48 - .54	.078 - .11 - .15	.062 - .094 - .12	.042 - .073 - .1	.036 - .062 - .094	.66 - .7 - .76	0 - .016 - .031
hmdh	.62 - .69 - .78	.24 - .3 - .38	.66 - .76 - .85	.4 - .48 - .57	.33 - .4 - .49	.067 - .11 - .18	.034 - .079 - .12	.034 - .079 - .12	1 - 1 - 1	0 - 0 - 0
hs90a	.1 - .2 - .29	.33 - .45 - .55	.041 - .12 - .2	.35 - .47 - .57	0 - .02 - .061	.16 - .27 - .39	0 - 0 - 0	0 - 0 - 0	.94 - .98 - 1	0 - 0 - 0
hxk4	.27 - .4 - .5	.25 - .35 - .48	.15 - .25 - .35	.29 - .42 - .54	0 - .021 - .062	.062 - .12 - .21	0 - .062 - .1	.021 - .062 - .12	.46 - .56 - .69	.042 - .12 - .19
igf1r	.39 - .48 - .56	.2 - .28 - .35	.32 - .4 - .49	.2 - .28 - .35	.21 - .29 - .37	.16 - .23 - .31	.18 - .26 - .32	.096 - .16 - .22	.65 - .73 - .81	0 - 0 - .011
inha	0 - .043 - .13	0 - 0 - 0	0 - 0 - 0	0 - 0 - .087	0 - .043 - .13	0 - 0 - 0	.043 - .13 - .26	.17 - .3 - .48	.87 - 1 - 1	0 - .043 - .087
ital	.047 - .1 - .15	.058 - .1 - .16	.023 - .07 - .13	.047 - .1 - .15	.035 - .07 - .12	.058 - .12 - .17	0 - 0 - 0	.023 - .058 - .1	.93 - .98 - 1	0 - 0 - 0
jak2	.36 - .45 - .56	.42 - .52 - .62	.27 - .36 - .45	.53 - .65 - .74	.3 - .39 - .48	.32 - .42 - .52	.18 - .26 - .35	.24 - .32 - .41	.76 - .85 - .94	.11 - .18 - .26
kif11	.53 - .63 - .71	.29 - .37 - .47	.51 - .61 - .71	.47 - .59 - .7	.34 - .43 - .53	.014 - .043 - .086	.33 - .4 - .5	.14 - .21 - .3	.94 - .97 - 1	.014 - .043 - .1
kit	.15 - .22 - .27	.085 - .13 - .19	.1 - .16 - .23	.057 - .1 - .15	.19 - .25 - .32	.066 - .1 - .17	.028 - .075 - .11	.094 - .15 - .21	.62 - .71 - .76	.16 - .24 - .29
kith	.59 - .76 - .86	.52 - .69 - .83	.17 - .31 - .48	.62 - .72 - .86	.14 - .28 - .41	.21 - .34 - .48	.45 - .66 - .83	.14 - .24 - .38	.93 - 1 - 1	0 - 0 - 0
kpcb	.27 - .36 - .44	.44 - .53 - .61	.25 - .34 - .43	.45 - .55 - .62	.27 - .35 - .42	.22 - .27 - .36	.35 - .43 - .52	.16 - .23 - .31	.82 - .88 - .93	0 - .011 - .034
lck	.19 - .23 - .27	.32 - .36 - .41	.18 - .22 - .26	.32 - .36 - .41	.25 - .29 - .34	.27 - .32 - .36	.12 - .15 - .18	.11 - .14 - .17	.82 - .86 - .89	.2 - .24 - .28
lkha4	.21 - .28 - .35	.43 - .5 - .57	.2 - .26 - .33	.35 - .43 - .51	.3 - .39 - .47	.29 - .36 - .45	.18 - .26 - .33	.19 - .25 - .32	.95 - .99 - 1	.062 - .11 - .17
mapk2	.19 - .27 - .37	.34 - .45 - .55	.4 - .5 - .6	.34 - .44 - .53	.19 - .27 - .37	.31 - .42 - .5	.15 - .23 - .32	.32 - .44 - .52	.79 - .87 - .95	.016 - .048 - .11
mcr	.12 - .19 - .29	0 - .019 - .058	.15 - .25 - .33	.13 - .21 - .33	.019 - .077 - .15	0 - .019 - .077	.058 - .13 - .21	0 - .019 - .058	.75 - .85 - .92	.019 - .077 - .15
met	.47 - .55 - .64	.29 - .37 - .45	.46 - .54 - .6	.46 - .54 - .63	.47 - .55 - .62	.29 - .36 - .43	.11 - .18 - .23	.24 - .32 - .39	.84 - .91 - .96	.035 - .07 - .11
mk01	.087 - .17 - .28	.11 - .22 - .3	.13 - .22 - .3	.13 - .24 - .35	.087 - .2 - .3	.087 - .17 - .24	0 - .043 - .11	.15 - .26 - .35	.65 - .74 - .87	0 - .022 - .065
mk10	.045 - .12 - .18	.045 - .1 - .18	.09 - .16 - .22	.09 - .16 - .22	.12 - .19 - .27	.06 - .12 - .18	.06 - .12 - .18	.19 - .27 - .36	.48 - .58 - .67	0 - 0 - 0
mk14	.27 - .31 - .35	.2 - .24 - .27	.3 - .34 - .38	.34 - .38 - .42	.2 - .24 - .28	.25 - .29 - .33	.08 - .1 - .13	.1 - .13 - .16	.79 - .82 - .87	.16 - .2 - .23
mmp13	.11 - .14 - .17	.21 - .24 - .28	.23 - .27 - .3	.44 - .48 - .52	.2 - .24 - .27	.21 - .25 - .28	.04 - .061 - .082	.085 - .11 - .14	.92 - .94 - .96	.034 - .056 - .074
mp2k1	.036 - .084 - .14	.048 - .11 - .17	.012 - .048 - .096	.036 - .072 - .12	.036 - .072 - .12	.096 - .16 - .22	0 - 0 - 0	.06 - .12 - .18	.024 - .072 - .12	0 - .012 - .036
nos1	.049 - .098 - .16	.085 - .15 - .21	.037 - .073 - .12	.049 - .11 - .16	0 - .037 - .073	.024 - .061 - .11	0 - .024 - .061	.024 - .061 - .11	.59 - .67 - .74	0 - .012 - .037
nram	.19 - .27 - .35	.41 - .51 - .62	.21 - .29 - .4	.38 - .49 - .59	.24 - .33 - .41	.13 - .21 - .3	0 - 0 - 0	0 - 0 - 0	.84 - .92 - .97	0 - 0 - 0
pa2ga	.077 - .17 - .25	.5 - .63 - .75	.12 - .19 - .29	.5 - .63 - .71	.096 - .17 - .27	.23 - .31 - .42	0 - .019 - .058	0 - .038 - .096	.9 - .96 - 1	.13 - .21 - .31
parp1	.23 - .27 - .31	.58 - .62 - .68	.31 - .36 - .4	.75 - .79 - .83	.21 - .25 - .29	.29 - .33 - .37	.19 - .23 - .26	.22 - .26 - .3	.92 - .94 - .96	.089 - .12 - .15
pde5a	.16 - .2 - .24	.19 - .23 - .27	.23 - .27 - .32	.28 - .32 - .36	.05 - .075 - .1	.057 - .086 - .11	.13 - .17 - .2	.11 - .14 - .17	.039 - .061 - .086	.0036 - .014 - .025
pgh1	.12 - .16 - .23	.027 - .064 - .1	.11 - .15 - .23	.073 - .12 - .16	.091 - .15 - .2	.027 - .064 - .11	.064 - .11 - .15	.0091 - .027 - .055	.85 - .92 - .96	0 - .0091 - .045
pgh2	.26 - .32 - .36	.21 - .26 - .31	.19 - .24 - .28	.19 - .23 - .28	.17 - .22 - .26	.1 - .14 - .17	.42 - .48 - .53	.2 - .25 - .3	.93 - .95 - .97	.025 - .051 - .076
plk1	.51 - .59 - .72	.51 - .62 - .72	.52 - .61 - .7	.51 - .59 - .71	.41 - .49 - .59	.32 - .42 - .51	0 - 0 - 0	.13 - .2 - .28	.029 - .072 - .13	0 - .014 - .043
pnph	.41 - .51 - .59	.55 - .66 - .75	.18 - .28 - .35	.34 - .42 - .51	.27 - .35 - .44	.13 - .21 - .3	.099 - .17 - .25	.2 - .28 - .38	.97 - 1 - 1	.028 - .099 - .14
ppara	.056 - .086 - .13	.041 - .066 - .096	.12 - .17 - .2	.26 - .3 - .36	.0051 - .015 - .03	.0051 - .02 - .041	.086 - .13 - .16	.18 - .23 - .28	.81 - .85 - .89	.03 - .051 - .076
ppard	.048 - .088 - .13	.14 - .19 - .25	.072 - .12 - .17	.15 - .21 - .26	0 - .008 - .024	.008 - .032 - .064	0 - .024 - .048	.048 - .088 - .14	.95 - .98 - 1	.1 - .16 - .21
pparg	.082 - .11 - .15	.097 - .13 - .16	.093 - .13 - .16	.17 - .21 - .25	.047 - .066 - .097	.062 - .089 - .12	.086 - .12 - .15	.14 - .18 - .21	.75 - .79 - .83	.11 - .14 - .18
prgr	.27 - .34 - .4	.31 - .36 - .44	.37 - .43 - .49	.48 - .55 - .6	.1 - .14 - .19	.17 - .22 - .27	.18 - .23 - .29	.025 - .057 - .088	.93 - .96 - .99	.069 - .11 - .15
ptn1	.34 - .45 - .55	.3 - .38 - .47	.32 - .43 - .5	.36 - .46 - .55	.31 - .41 - .49	.18 - .26 - .34	.36 - .46 - .55	.45 - .55 - .65	.82 - .88 - .95	.19 - .28 - .36
pur2	.81 - .93 - 1	1 - 1 - 1	.7 - .81 - .93	.89 - .96 - 1	.78 - .85 - .96	.89 - .96 - 1	0 - .037 - .15	.59 - .74 - .85	.85 - .93 - 1	0 - 0 - 0
pygm	.025 - .075 - .15	0 - 0 - 0	.025 - .075 - .15	0 - 0 - 0	0 - .05 - .12	0 - .025 - .05	0 - .025 - .1	.025 - .1 - .17	.85 - .95 - 1	0 - .025 - .075
pyrd	.27 - .38 - .47	.24 - .35 - .44	.3 - .41 - .5	.47 - .56 - .65	.14 - .21 - .29	.045 - .11 - .17	.26 - .35 - .42	.076 - .14 - .2	.89 - .94 - .98	.061 - .14 - .2
reni	.056 - .13 - .18	.2 - .27 - .35	.11 - .2 - .25	.23 - .3 - .38	.014 - .042 - .085	.14 - .21 - .28	.028 - .07 - .13	.11 - .2 - .25	.32 - .42 - .51	.14 - .21 - .3
rock1	.2 - .3 - .39	.31 - .41 - .5	.27 - .36 - .45	.42 - .53 - .62	.094 - .17 - .23	.17 - .27 - .36	.047 - .11 - .17	.062 - .12 - .19	.8 - .88 - .94	.031 - .11 - .16
rxra	.55 - .66 - .75	.14 - .21 - .3	.63 - .7 - .79	.48 - .58 - .66	.056 - .099 - .17	.014 - .056 - .11	.46 - .55 - .65	.2 - .28 - .38	1 - 1 - 1	.014 - .056 - .099
sahh	0 - 0 - 0	.31 - .46 - .6	.057 - .14 - .26	.17 - .29 - .4	0 - .029 - .086	0 - .029 - .086	.26 - .37 - .51	.17 - .29 - .4	.74 - .86 - .94	0 - .057 - .11
src	.15 - .18 - .21	.35 - .39 - .43	.21 - .25 - .28	.27 - .31 - .35	.33 - .36 - .4	.22 - .25 - .29	.054 - .083 - .1	.094 - .12 - .15	.81 - .85 - .89	.02 - .034 - .051
tgfr1	.36 - .44 - .51	.33 - .42 - .5	.53 - .62 - .69	.59 - .67 - .76	.29 - .37 - .44	.43 - .51 - .6	.093 - .16 - .22	.28 - .36 - .44	.86 - .92 - .99	0 - 0 - 0
thb	.4 - .48 - .57	.56 - .65 - .75	.47 - .56 - .64	.36 - .45 - .55	.04 - .093 - .13	.027 - .08 - .13	.29 - .36 - .44	.36 - .45 - .55	.96 - .99 - 1	.067 - .12 - .19
thrb	.54 - .59 - .64	.54 - .59 - .64	.47 - .52 - .56	.57 - .62 - .67	.54 - .59 - .63	.5 - .55 - .6	.011 - .026 - .044	.19 - .24 - .27	.89 - .92 - .95	.12 - .15 - .19
try1	.35 - .39 - .44	.34 - .39 - .44	.28 - .33 - .38	.27 - .32 - .36	.34 - .39 - .43	.27 - .32 - .36	.027 - .045 - .064	.087 - .12 - .16	.83 - .88 - .9	.091 - .13 - .16
tryb1	.013 - .038 - .077	.077 - .14 - .22	.013 - .064 - .12	.09 - .15 - .23	.051 - .12 - .18	.051 - .1 - .17	.09 - .15 - .23	.15 - .23 - .31	.77 - .85 - .91	.064 - .14 - .19
tysy	.38 - .49 - .57	.1 - .18 - .26	.32 - .41 - .51	.044 - .088 - .18	.34 - .44 - .53	.029 - .074 - .15	.28 - .37 - .46	.47 - .57 - .65	.75 - .85 - .93	.059 - .12 - .19
urok	.14 - .2 - .27	.26 - .34 - .4	.05 - .1 - .15	.15 - .22 - .28	.23 - .31 - .38	.23 - .31 - .38	.08 - .13 - .18	.12 - .18 - .24	.77 - .84 - .89	0 - .01 - .04
vgfr2	.32 - .37 - .41	.18 - .22 - .26	.32 - .37 - .41	.36 - .41 - .47	.3 - .34 - .39	.18 - .22 - .27	.24 - .28 - .32	.26 - .31 - .35	.81 - .86 - .9	.04 - .063 - .087
wee1	.9 - .95 - 1	.95 - .98 - 1	.68 - .77 - .85	.81 - .87 - .94	.9 - .97 - 1	.89 - .94 - 1	.82 - .89 - .97	.58 - .71 - .82	1 - 1 - 1	.73 - .81 - .89
xiap	.56 - .67 - .79	.77 - .87 - .92	.44 - .54 - .65	.58 - .69 - .79	.29 - .4 - .5	.73 - .83 - .96	.077 - .19 - .25	0 - 0 - 0	.94 - .98 - 1	0 - 0 - 0

Difference from Default (Affinity)

-0.2 -0.1 0.0 0.1 0.2

Figure S11: NEF1% performance, with 5%–95% confidence intervals, shaded by difference with Default (Affinity). White boxes are not significant (p > 0.0005). Red means the method is better than Default (Affinity) and blue worse.

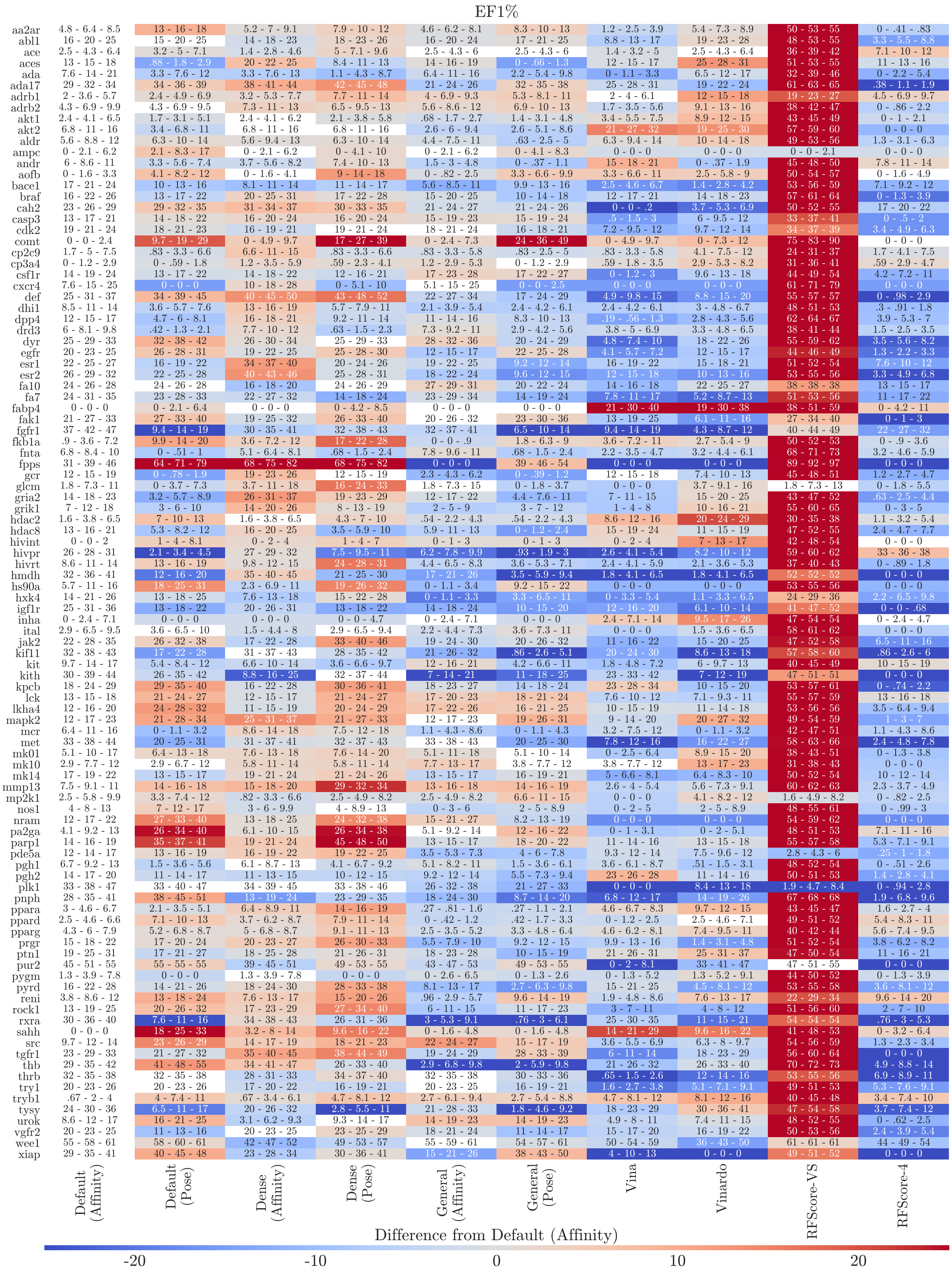

Figure S12: DUD-E EF1% performance, with 5%–95% confidence intervals, shaded by difference with Default (Affinity). White boxes are not significant (p > 0.0005). Red means the method is better than Default (Affinity) and blue worse.

AUC

	Default (Affinity)	Default (Pose)	Dense (Affinity)	Dense (Pose)	General (Affinity)	General (Pose)	Vina	Vinardo	RFScore-VS	RFScore-4
aa2ar	.72 - .74 - .76	.8 - .82 - .84	.75 - .77 - .79	.76 - .77 - .79	.61 - .63 - .65	.81 - .83 - .84	.63 - .65 - .67	.82 - .83 - .85	.93 - .94 - .95	.62 - .64 - .66
abl1	.76 - .79 - .81	.62 - .66 - .7	.74 - .77 - .8	.62 - .66 - .71	.71 - .74 - .77	.61 - .65 - .69	.75 - .78 - .81	.8 - .83 - .86	.95 - .96 - .97	.71 - .74 - .77
ace	.59 - .61 - .64	.59 - .62 - .65	.57 - .59 - .62	.58 - .61 - .64	.53 - .56 - .59	.52 - .55 - .58	.54 - .57 - .6	.56 - .59 - .62	.93 - .94 - .95	.6 - .63 - .66
aces	.6 - .62 - .65	.62 - .64 - .66	.6 - .63 - .65	.62 - .65 - .67	.63 - .66 - .68	.56 - .59 - .61	.76 - .78 - .8	.73 - .75 - .77	.94 - .95 - .96	.59 - .61 - .64
ada	.72 - .76 - .8	.79 - .83 - .86	.72 - .75 - .79	.68 - .72 - .76	.71 - .75 - .79	.84 - .86 - .89	.52 - .57 - .62	.7 - .73 - .77	.93 - .94 - .96	.64 - .68 - .72
ada17	.8 - .82 - .84	.84 - .86 - .88	.85 - .87 - .89	.86 - .88 - .89	.76 - .78 - .81	.83 - .85 - .87	.69 - .71 - .73	.74 - .76 - .78	.96 - .97 - .98	.55 - .57 - .59
adrb1	.6 - .63 - .66	.63 - .66 - .69	.61 - .64 - .67	.62 - .66 - .69	.66 - .7 - .73	.65 - .68 - .71	.71 - .73 - .76	.76 - .79 - .81	.9 - .91 - .93	.62 - .66 - .69
adrb2	.67 - .71 - .74	.71 - .74 - .77	.72 - .75 - .78	.74 - .77 - .79	.7 - .73 - .76	.7 - .73 - .76	.68 - .71 - .74	.72 - .74 - .77	.94 - .95 - .96	.58 - .61 - .64
akt1	.74 - .76 - .79	.47 - .49 - .52	.71 - .73 - .76	.51 - .54 - .57	.65 - .67 - .69	.44 - .47 - .5	.74 - .76 - .78	.71 - .74 - .76	.96 - .97 - .98	.67 - .69 - .71
akt2	.77 - .81 - .84	.39 - .44 - .49	.79 - .82 - .85	.47 - .52 - .57	.67 - .71 - .75	.41 - .45 - .5	.75 - .79 - .83	.75 - .79 - .83	.95 - .97 - .99	.73 - .76 - .8
aldr	.52 - .56 - .61	.61 - .65 - .69	.53 - .58 - .62	.61 - .65 - .69	.5 - .54 - .59	.59 - .62 - .66	.71 - .74 - .77	.67 - .71 - .75	.94 - .96 - .98	.56 - .6 - .64
ampc	.45 - .52 - .58	.71 - .76 - .81	.43 - .49 - .56	.7 - .75 - .8	.42 - .49 - .55	.54 - .6 - .65	.55 - .61 - .67	.54 - .6 - .66	.076 - .13 - .19	.37 - .43 - .49
andr	.77 - .79 - .81	.56 - .58 - .61	.77 - .8 - .82	.66 - .69 - .72	.73 - .75 - .77	.44 - .47 - .5	.59 - .63 - .66	.54 - .57 - .6	.96 - .97 - .97	.69 - .72 - .74
aofb	.46 - .51 - .55	.64 - .69 - .73	.49 - .53 - .57	.66 - .7 - .75	.4 - .44 - .47	.56 - .61 - .66	.75 - .78 - .82	.73 - .76 - .8	.96 - .98 - .98	.51 - .55 - .59
bace1	.8 - .82 - .84	.68 - .71 - .74	.77 - .79 - .81	.69 - .72 - .75	.69 - .71 - .74	.64 - .67 - .7	.7 - .72 - .75	.71 - .73 - .76	.95 - .96 - .97	.72 - .74 - .76
braf	.81 - .83 - .86	.6 - .65 - .69	.82 - .85 - .87	.61 - .66 - .7	.79 - .82 - .85	.58 - .63 - .67	.84 - .86 - .88	.81 - .84 - .87	.95 - .97 - .98	.72 - .75 - .78
cah2	.92 - .93 - .94	.89 - .91 - .92	.94 - .95 - .96	.9 - .91 - .92	.91 - .92 - .93	.87 - .88 - .89	.57 - .59 - .61	.67 - .69 - .71	.97 - .98 - .98	.79 - .81 - .83
casp3	.62 - .66 - .7	.75 - .78 - .81	.71 - .74 - .77	.76 - .79 - .82	.62 - .66 - .69	.72 - .76 - .78	.67 - .7 - .73	.61 - .64 - .68	.91 - .93 - .94	.43 - .47 - .5
cdk2	.78 - .8 - .82	.73 - .76 - .78	.78 - .8 - .82	.76 - .78 - .8	.72 - .74 - .76	.67 - .69 - .72	.7 - .72 - .74	.73 - .75 - .77	.9 - .91 - .92	.68 - .7 - .73
comt	.58 - .65 - .72	.93 - .94 - .96	.71 - .77 - .83	.95 - .96 - .98	.66 - .73 - .79	.93 - .95 - .97	.57 - .63 - .69	.67 - .72 - .77	1 - 1 - 1	.43 - .49 - .54
cp2c9	.57 - .62 - .66	.54 - .58 - .63	.58 - .63 - .67	.55 - .6 - .65	.55 - .59 - .63	.5 - .55 - .59	.58 - .62 - .67	.67 - .71 - .75	.87 - .89 - .92	.53 - .58 - .63
cp3a4	.62 - .65 - .69	.48 - .51 - .55	.62 - .65 - .69	.5 - .54 - .58	.6 - .64 - .67	.47 - .5 - .54	.57 - .61 - .64	.65 - .69 - .72	.91 - .92 - .94	.62 - .66 - .7
csf1r	.75 - .78 - .81	.68 - .72 - .76	.74 - .77 - .8	.67 - .71 - .75	.74 - .78 - .81	.67 - .71 - .75	.65 - .69 - .72	.74 - .77 - .8	.93 - .95 - .96	.75 - .78 - .81
cxcr4	.76 - .85 - .92	.59 - .66 - .73	.68 - .77 - .86	.68 - .75 - .81	.69 - .77 - .84	.58 - .64 - .71	.54 - .6 - .65	.49 - .56 - .62	.99 - 1 - 1	.65 - .71 - .76
def	.9 - .93 - .96	.91 - .94 - .97	.94 - .96 - .98	.92 - .95 - .98	.88 - .91 - .94	.89 - .92 - .96	.72 - .76 - .8	.71 - .76 - .8	.99 - 1 - 1	.54 - .58 - .62
dhi1	.7 - .72 - .75	.66 - .68 - .71	.71 - .74 - .76	.67 - .7 - .72	.68 - .7 - .72	.56 - .59 - .62	.75 - .77 - .79	.69 - .71 - .74	.94 - .95 - .96	.56 - .59 - .61
dpp4	.73 - .75 - .77	.65 - .66 - .68	.74 - .76 - .78	.64 - .66 - .69	.74 - .76 - .77	.7 - .72 - .74	.61 - .62 - .64	.58 - .6 - .62	.94 - .95 - .96	.66 - .67 - .69
drd3	.69 - .71 - .73	.64 - .66 - .67	.69 - .71 - .73	.56 - .58 - .59	.74 - .75 - .77	.68 - .7 - .71	.73 - .75 - .77	.65 - .68 - .7	.91 - .92 - .93	.65 - .67 - .69
dyr	.88 - .9 - .92	.82 - .85 - .88	.88 - .9 - .92	.79 - .82 - .85	.87 - .89 - .91	.82 - .85 - .88	.75 - .77 - .79	.85 - .87 - .89	.95 - .96 - .97	.77 - .79 - .81
egfr	.76 - .77 - .79	.8 - .81 - .83	.79 - .81 - .83	.79 - .81 - .83	.68 - .7 - .72	.74 - .76 - .78	.62 - .64 - .66	.75 - .76 - .78	.93 - .94 - .95	.62 - .64 - .66
esr1	.78 - .8 - .83	.7 - .73 - .75	.82 - .84 - .86	.71 - .74 - .77	.76 - .78 - .81	.71 - .73 - .75	.81 - .83 - .84	.8 - .82 - .84	.98 - .98 - .99	.73 - .75 - .77
esr2	.77 - .79 - .81	.71 - .74 - .77	.83 - .86 - .88	.75 - .78 - .81	.73 - .76 - .78	.67 - .69 - .72	.77 - .8 - .82	.76 - .78 - .8	.99 - .99 - .99	.72 - .74 - .76
fa10	.87 - .88 - .9	.83 - .85 - .87	.85 - .87 - .88	.84 - .86 - .87	.89 - .9 - .92	.8 - .81 - .83	.82 - .83 - .85	.8 - .82 - .83	.99 - .99 - .99	.85 - .86 - .88
fa7	.89 - .91 - .93	.86 - .89 - .91	.93 - .94 - .95	.88 - .9 - .93	.89 - .91 - .92	.77 - .81 - .85	.89 - .91 - .93	.82 - .85 - .87	.99 - .99 - 1	.84 - .87 - .89
fabp4	.46 - .53 - .59	.67 - .72 - .77	.41 - .47 - .53	.63 - .68 - .73	.42 - .48 - .53	.55 - .62 - .69	.71 - .78 - .85	.81 - .85 - .89	.92 - .94 - .97	.49 - .56 - .63
fak1	.81 - .84 - .87	.63 - .68 - .73	.72 - .77 - .81	.52 - .59 - .64	.66 - .71 - .75	.59 - .65 - .71	.77 - .81 - .84	.77 - .8 - .83	.96 - .97 - .98	.82 - .85 - .88
fgfr1	.92 - .94 - .96	.56 - .62 - .68	.93 - .94 - .96	.69 - .74 - .78	.93 - .94 - .96	.51 - .57 - .63	.71 - .74 - .78	.75 - .77 - .8	.93 - .94 - .96	.88 - .91 - .93
fkb1a	.74 - .78 - .81	.81 - .84 - .87	.71 - .74 - .78	.79 - .82 - .86	.74 - .77 - .8	.78 - .81 - .84	.74 - .77 - .8	.63 - .67 - .71	.94 - .96 - .98	.64 - .68 - .71
fnta	.77 - .78 - .8	.54 - .55 - .57	.69 - .71 - .73	.5 - .52 - .54	.79 - .8 - .82	.55 - .57 - .59	.69 - .71 - .73	.76 - .77 - .79	.82 - .84 - .87	.72 - .74 - .75
fpps	.97 - .98 - .98	.95 - .97 - .99	.97 - .98 - .99	.94 - .96 - .98	.88 - .89 - .9	.9 - .93 - .95	.25 - .29 - .32	.63 - .66 - .69	1 - 1 - 1	.37 - .39 - .41
gcr	.72 - .75 - .78	.58 - .61 - .64	.77 - .8 - .83	.7 - .73 - .76	.68 - .71 - .74	.51 - .54 - .57	.6 - .64 - .68	.63 - .66 - .68	.92 - .94 - .95	.62 - .65 - .68
glcm	.51 - .58 - .65	.65 - .71 - .78	.63 - .7 - .77	.79 - .85 - .89	.52 - .59 - .66	.59 - .65 - .72	.44 - .49 - .55	.58 - .65 - .72	.74 - .78 - .83	.47 - .55 - .63
gria2	.8 - .83 - .86	.69 - .73 - .76	.82 - .85 - .88	.72 - .77 - .81	.78 - .81 - .84	.66 - .69 - .73	.71 - .75 - .78	.81 - .84 - .87	.94 - .96 - .97	.56 - .6 - .63
grik1	.7 - .73 - .77	.75 - .79 - .82	.8 - .83 - .86	.82 - .86 - .89	.64 - .68 - .72	.71 - .75 - .8	.54 - .59 - .65	.73 - .77 - .81	.99 - .99 - 1	.46 - .51 - .55
hdac2	.79 - .8 - .82	.83 - .85 - .87	.81 - .82 - .84	.83 - .85 - .87	.75 - .77 - .79	.75 - .78 - .8	.82 - .85 - .87	.81 - .84 - .87	.92 - .94 - .95	.78 - .8 - .82
hdac8	.8 - .83 - .86	.74 - .77 - .81	.83 - .86 - .89	.75 - .79 - .82	.75 - .78 - .81	.63 - .67 - .7	.79 - .82 - .85	.75 - .79 - .82	.95 - .96 - .97	.69 - .72 - .75
hivint	.45 - .5 - .54	.57 - .62 - .66	.53 - .58 - .62	.56 - .61 - .65	.44 - .49 - .54	.52 - .56 - .6	.67 - .71 - .74	.81 - .84 - .87	.95 - .96 - .98	.44 - .49 - .53
hivpr	.83 - .85 - .86	.77 - .78 - .8	.84 - .86 - .88	.79 - .81 - .82	.74 - .76 - .78	.65 - .66 - .68	.7 - .72 - .74	.79 - .81 - .82	.96 - .97 - .98	.76 - .78 - .8
hivrt	.79 - .81 - .83	.73 - .76 - .78	.79 - .82 - .84	.77 - .79 - .82	.71 - .73 - .75	.64 - .66 - .69	.66 - .68 - .71	.57 - .6 - .62	.89 - .91 - .93	.55 - .58 - .6
hmdh	.9 - .92 - .94	.87 - .89 - .9	.9 - .92 - .95	.88 - .9 - .92	.85 - .88 - .9	.8 - .82 - .84	.76 - .79 - .81	.68 - .71 - .73	.98 - .98 - .99	.62 - .65 - .68
hs90a	.68 - .73 - .77	.76 - .81 - .86	.68 - .72 - .77	.78 - .83 - .87	.67 - .71 - .75	.72 - .78 - .83	.23 - .26 - .29	.53 - .57 - .61	.97 - .98 - .99	.45 - .49 - .54
hxk4	.78 - .82 - .86	.69 - .74 - .78	.75 - .79 - .83	.66 - .71 - .76	.68 - .72 - .77	.63 - .68 - .72	.53 - .57 - .61	.68 - .72 - .76	.88 - .91 - .94	.66 - .71 - .75
igf1r	.83 - .85 - .88	.54 - .58 - .63	.77 - .8 - .84	.57 - .61 - .66	.83 - .86 - .88	.5 - .55 - .59	.81 - .83 - .86	.81 - .84 - .86	.96 - .97 - .98	.68 - .71 - .73
inha	.61 - .68 - .73	.6 - .66 - .72	.57 - .64 - .69	.58 - .65 - .71	.65 - .71 - .77	.56 - .62 - .68	.66 - .71 - .78	.85 - .89 - .92	.91 - .95 - .98	.59 - .67 - .74
ital	.62 - .66 - .69	.49 - .53 - .57	.6 - .64 - .68	.46 - .51 - .55	.52 - .56 - .6	.44 - .49 - .52	.56 - .6 - .63	.55 - .59 - .63	.97 - .98 - .99	.62 - .65 - .69
jak2	.89 - .91 - .94	.86 - .89 - .92	.9 - .92 - .94	.87 - .9 - .92	.82 - .86 - .89	.83 - .87 - .9	.73 - .78 - .81	.84 - .87 - .89	.96 - .97 - .98	.77 - .81 - .85
kif11	.76 - .81 - .85	.81 - .84 - .87	.81 - .85 - .89	.81 - .85 - .87	.76 - .81 - .85	.77 - .8 - .83	.82 - .85 - .89	.7 - .74 - .78	.96 - .97 - .99	.69 - .73 - .77
kit	.77 - .79 - .82	.54 - .58 - .62	.76 - .79 - .81	.62 - .66 - .69	.78 - .8 - .83	.54 - .58 - .63	.76 - .78 - .8	.74 - .77 - .79	.92 - .94 - .96	.77 - .8 - .83
kith	.65 - .73 - .79	.73 - .8 - .87	.63 - .7 - .77	.73 - .8 - .87	.61 - .69 - .76	.72 - .79 - .84	.67 - .74 - .8	.71 - .77 - .82	.88 - .92 - .96	.51 - .57 - .64
kpcb	.67 - .71 - .76	.72 - .76 - .8	.68 - .73 - .77	.69 - .74 - .79	.71 - .75 - .79	.73 - .77 - .81	.73 - .77 - .82	.71 - .75 - .79	.94 - .96 - .97	.66 - .7 - .74
lck	.74 - .76 - .78	.7 - .73 - .75	.74 - .76 - .78	.7 - .73 - .75	.74 - .77 - .79	.69 - .72 - .75	.78 - .8 - .81	.75 - .77 - .79	.97 - .98 - .98	.74 - .76 - .78
lkha4	.85 - .88 - .9	.9 - .92 - .94	.88 - .9 - .92	.89 - .91 - .93	.81 - .84 - .86	.83 - .86 - .88	.87 - .89 - .92	.87 - .89 - .91	.95 - .97 - .98	.65 - .68 - .72
mapk2	.88 - .9 - .92	.8 - .84 - .88	.94 - .95 - .96	.82 - .86 - .89	.83 - .86 - .88	.78 - .82 - .86	.86 - .89 - .91	.84 - .87 - .9	.98 - .99 - .99	.71 - .75 - .78
mcr	.8 - .84 - .87	.54 - .57 - .61	.81 - .84 - .88	.65 - .69 - .73	.77 - .81 - .84	.46 - .5 - .54	.49 - .54 - .6	.46 - .51 - .56	.91 - .94 - .96	.73 - .77 - .82
met	.85 - .88 - .9	.78 - .81 - .84	.85 - .87 - .9	.78 - .82 - .85	.83 - .86 - .89	.69 - .73 - .77	.78 - .81 - .84	.85 - .87 - .89	.96 - .97 - .98	.71 - .74 - .77
mk01	.72 - .76 - .81	.55 - .61 - .67	.7 - .75 - .8	.55 - .61 - .68	.71 - .75 - .79	.54 - .61 - .66	.81 - .86 - .89	.76 - .81 - .86	.93 - .96 - .98	.69 - .74 - .79
mk10	.77 - .81 - .84	.69 - .73 - .77	.78 - .81 - .85	.72 - .76 - .8	.76 - .8 - .84	.68 - .72 - .76	.71 - .75 - .79	.77 - .81 - .84	.91 - .92 - .94	.64 - .68 - .72
mk14	.73 - .75 - .76	.6 - .63 - .65	.74 - .76 - .78	.63 - .65 - .67	.69 - .71 - .73	.59 - .61 - .63	.72 - .74 - .76	.78 - .8 - .81	.95 - .96 - .97	.74 - .76 - .78
mmp13	.73 - .75 - .77	.78 - .8 - .82	.78 - .79 - .81	.82 - .84 - .86	.76 - .78 - .8	.75 - .77 - .79	.63 - .66 - .68	.66 - .68 - .7	.96 - .97 - .98	.58 - .6 - .62
mp2k1	.66 - .7 - .74	.52 - .57 - .62	.68 - .72 - .75	.55 - .59 - .63	.6 - .65 - .69	.53 - .58 - .62	.5 - .53 - .57	.68 - .72 - .76	.63 - .67 - .71	.49 - .53 - .57
nos1	.59 - .64 - .7	.72 - .77 - .82	.57 - .62 - .67	.71 - .76 - .8	.56 - .61 - .66	.68 - .73 - .77	.54 - .59 - .64	.65 - .7 - .75	.92 - .94 - .97	.51 - .56 - .61
nram	.92 - .94 - .95	.92 - .94 - .95	.95 - .96 - .97	.91 - .93 - .95	.92 - .94 - .95	.86 - .88 - .91	.5 - .54 - .58	.35 - .39 - .43	.97 - .98 - .99	.58 - .61 - .64
pa2ga	.77 - .81 - .84	.84 - .88 - .91	.8 - .84 - .87	.79 - .83 - .88	.74 - .78 - .81	.86 - .89 - .91	.58 - .62 - .66	.72 - .76 - .8	.96 - .98 - .99	.72 - .77 - .81
parp1	.86 - .87 - .88	.93 - .93 - .94	.9 - .91 - .92	.94 - .95 - .96	.88 - .89 - .9	.86 - .88 - .89	.85 - .86 - .87	.87 - .88 - .89	.98 - .99 - .99	.69 - .7 - .72
pde5a	.79 - .81 - .82	.8 - .81 - .83	.86 - .88 - .89	.84 - .85 - .87	.72 - .74 - .76	.72 - .74 - .76	.64 - .66 - .69	.76 - .78 - .8	.72 - .74 - .76	.65 - .67 - .69
pgh1	.64 - .67 - .71	.62 - .65 - .68	.64 - .67 - .71	.63 - .67 - .7	.61 - .65 - .68	.56 - .6 - .63	.61 - .64 - .68	.61 - .64 - .67	.91 - .93 - .95	.52 - .55 - .58
pgh2	.73 - .75 - .77	.7 - .73 - .75	.74 - .76 - .78	.72 - .75 - .77	.69 - .72 - .74	.65 - .67 - .69	.75 - .77 - .8	.69 - .71 - .73	.94 - .95 - .96	.52 - .54 - .55
plk1	.88 - .91 - .93	.88 - .91 - .93	.91 - .93 - .95	.89 - .92 - .94	.83 - .86 - .9	.83 - .87 - .91	.59 - .64 - .69	.76 - .8 - .83	.68 - .72 - .77	.63 - .67 - .71
pnph	.89 - .91 - .94	.79 - .84 - .88	.84 - .87 - .9	.78 - .83 - .87	.78 - .82 - .86	.68 - .73 - .78	.86 - .88 - .9	.88 - .9 - .92	.99 - 1 - 1	.52 - .57 - .61
ppara	.73 - .76 - .78	.73 - .75 - .77	.75 - .77 - .79	.74 - .76 - .79	.59 - .62 - .64	.63 - .65 - .67	.85 - .86 - .88	.78 - .8 - .82	.96 - .97 - .98	.65 - .68 - .7
ppard	.71 - .74 - .76	.67 - .7 - .73	.71 - .73 - .76	.65 - .68 - .72	.6 - .63 - .65	.63 - .65 - .68	.74 - .76 - .78	.64 - .67 - .7	.97 - .98 - .99	.65 - .68 - .71
pparg	.72 - .74 - .75	.71 - .73 - .75	.75 - .77 - .78	.73 - .75 - .77	.66 - .68 - .7	.66 - .68 - .7	.78 - .79 - .81	.72 - .74 - .77	.95 - .95 - .96	.75 - .77 - .78
prgr	.77 - .8 - .82	.7 - .73 - .75	.84 - .86 - .88	.84 - .86 - .88	.74 - .77 - .79	.6 - .63 - .66	.64 - .67 - .7	.58 - .61 - .64	.95 - .96 - .97	.69 - .72 - .74
ptn1	.78 - .81 - .85	.79 - .82 - .85	.79 - .82 - .85	.8 - .83 - .86	.74 - .78 - .82	.79 - .82 - .85	.8 - .83 - .87	.86 - .89 - .91	.97 - .98 - .99	.69 - .73 - .77
pur2	1 - 1 - 1	1 - 1 - 1	.99 - .99 - 1	.99 - 1 - 1	.98 - .99 - 1	.99 - 1 - 1	.88 - .91 - .93	.79 - .85 - .91	.99 - 1 - 1	.73 - .77 - .81
pygm	.45 - .51 - .56	.6 - .65 - .7	.52 - .57 - .62	.61 - .65 - .7	.53 - .58 - .64	.55 - .61 - .67	.54 - .6 - .66	.56 - .62 - .67	.95 - .97 - .98	.48 - .53 - .59
pyrd	.69 - .73 - .77	.62 - .68 - .73	.72 - .76 - .8	.69 - .74 - .79	.66 - .7 - .74	.57 - .62 - .68	.8 - .83 - .86	.79 - .82 - .85	.97 - .98 - .99	.7 - .74 - .77
reni	.71 - .75 - .79	.69 - .74 - .78	.73 - .77 - .81	.71 - .75 - .8	.58 - .63 - .68	.57 - .62 - .68	.62 - .66 - .71	.63 - .68 - .72	.91 - .93 - .95	.79 - .83 - .87
rock1	.82 - .85 - .87	.67 - .73 - .79	.83 - .86 - .89	.72 - .77 - .83	.72 - .76 - .8	.63 - .69 - .74	.69 - .72 - .75	.78 - .8 - .83	.97 - .98 - .99	.69 - .73 - .76
rxra	.91 - .93 - .95	.82 - .85 - .87	.95 - .97 - .98	.95 - .96 - .98	.77 - .8 - .83	.56 - .61 - .66	.76 - .81 - .85	.53 - .57 - .61	.99 - .99 - 1	.54 - .58 - .63
sahh	.71 - .75 - .79	.79 - .84 - .88	.71 - .75 - .79	.84 - .88 - .91	.55 - .6 - .65	.61 - .65 - .7	.74 - .8 - .86	.64 - .69 - .74	.92 - .95 - .98	.6 - .65 - .7
src	.82 - .84 - .86	.86 - .88 - .89	.83 - .85 - .86	.85 - .87 - .88	.82 - .84 - .85	.8 - .82 - .84	.63 - .65 - .67	.79 - .81 - .82	.97 - .97 - .98	.71 - .73 - .75
tgfr1	.91 - .93 - .94	.91 - .93 - .95	.94 - .96 - .97	.92 - .94 - .96	.87 - .89 - .91	.91 - .92 - .94	.88 - .9 - .92	.88 - .9 - .92	.99 - .99 - .99	.63 - .67 - .71
thb	.85 - .88 - .91	.85 - .89 - .92	.87 - .9 - .93	.84 - .88 - .91	.73 - .77 - .81	.71 - .75 - .8	.78 - .82 - .86	.77 - .81 - .85	.98 - .99 - 1	.55 - .6 - .65
thrb	.91 - .92 - .93	.87 - .88 - .9	.91 - .92 - .93	.87 - .89 - .9	.9 - .91 - .93	.85 - .86 - .88	.75 - .77 - .78	.78 - .8 - .82	.97 - .98 - .99	.78 - .8 - .81
try1	.88 - .89 - .91	.81 - .83 - .85	.87 - .88 - .9	.8 - .81 - .83	.85 - .87 - .88	.79 - .82 - .83	.78 - .8 - .81	.75 - .77 - .78	.97 - .98 - .98	.78 - .8 - .81
tryb1	.63 - .66 - .7	.69 - .73 - .76	.71 - .74 - .77	.72 - .75 - .78	.67 - .7 - .74	.73 - .76 - .79	.67 - .71 - .74	.68 - .72 - .76	.92 - .94 - .96	.69 - .73 - .76
tysy	.84 - .87 - .91	.71 - .75 - .79	.83 - .87 - .9	.75 - .78 - .82	.79 - .83 - .87	.69 - .73 - .77	.83 - .87 - .9	.86 - .89 - .92	.95 - .97 - .98	.73 - .78 - .82
urok	.79 - .82 - .84	.83 - .87 - .89	.74 - .77 - .8	.81 - .84 - .87	.83 - .86 - .88	.85 - .88 - .91	.74 - .77 - .8	.76 - .79 - .82	.97 - .98 - .99	.69 - .72 - .75
vgfr2	.81 - .83 - .84	.65 - .68 - .7	.83 - .85 - .86	.69 - .72 - .74	.78 - .8 - .82	.68 - .71 - .73	.75 - .77 - .79	.82 - .84 - .85	.95 - .96 - .97	.75 - .77 - .79
wee1	.95 - .96 - .98	.98 - .99 - 1	.94 - .96 - .98	.97 - .99 - 1	.98 - .98 - .99	.97 - .99 - 1	.94 - .96 - .97	.8 - .84 - .88	1 - 1 - 1	.83 - .88 - .91
xiap	.86 - .89 - .92	.89 - .92 - .95	.86 - .9 - .93	.88 - .91 - .93	.74 - .78 - .82	.86 - .89 - .92	.69 - .73 - .77	.52 - .56 - .6	.95 - .97 - .99	.63 - .67 - .7

Difference from Default (Affinity)

-0.2 -0.1 0.0 0.1 0.2

Figure S13: DUD-E AUC performance, with 5%–95% confidence intervals, shaded by difference with Default (Affinity). White boxes are not significant (p > 0.0005). Red means the method is better than Default (Affinity) and blue worse.

NEF1%

	Default (Affinity)	Default (Pose)	Dense (Affinity)	Dense (Pose)	General (Affinity)	General (Pose)	Vina	Vinardo	RFScore-VS	RFScore-4
ADRB2	0 - 0 - 0	.059 - .18 - .35	0 - 0 - 0	0 - .12 - .24	0 - .059 - .18	0 - .12 - .29	0 - 0 - 0	0 - 0 - 0	0 - 0 - 0	0 - 0 - 0
ALDH1	.08 - .093 - .1	.049 - .059 - .069	.077 - .09 - .1	.058 - .068 - .079	.077 - .089 - .1	.047 - .057 - .066	.066 - .077 - .087	.058 - .068 - .079	.062 - .073 - .083	.048 - .057 - .066
ESR1 ago	0 - .15 - .31	0 - 0 - 0	0 - .15 - .31	0 - .077 - .23	0 - .077 - .23	0 - 0 - 0	0 - .077 - .23	0 - 0 - 0	0 - .077 - .23	0 - .077 - .23
ESR1 ant	.04 - .12 - .18	0 - .06 - .12	.06 - .16 - .22	.02 - .08 - .14	.02 - .06 - .12	.02 - .06 - .12	.02 - .06 - .12	0 - .02 - .1	0 - 0 - .04	.02 - .06 - .14
FEN1	.0081 - .019 - .03	.011 - .024 - .038	.041 - .06 - .079	.076 - .1 - .12	.0054 - .014 - .024	.0054 - .016 - .027	.014 - .024 - .038	.0054 - .016 - .027	.016 - .03 - .043	.0054 - .014 - .024
GBA	.054 - .09 - .13	.018 - .042 - .066	.078 - .12 - .16	0 - .018 - .036	.024 - .054 - .084	.006 - .024 - .048	.012 - .036 - .06	.03 - .054 - .084	0 - 0 - .012	.018 - .036 - .06
IDH1	.051 - .13 - .23	0 - .026 - .077	.026 - .077 - .15	0 - .026 - .077	.077 - .15 - .26	0 - .026 - .077	0 - 0 - 0	0 - 0 - 0	0 - .077 - .15	0 - .026 - .077
KAT2A	.0052 - .015 - .031	0 - .0052 - .015	.01 - .026 - .046	0 - .01 - .021	0 - .01 - .026	0 - .0052 - .015	0 - .0052 - .015	0 - .01 - .026	.01 - .026 - .046	0 - .01 - .026
MAPK1	.0065 - .016 - .026	.0065 - .016 - .029	.0032 - .013 - .026	.0097 - .023 - .039	0 - .0097 - .019	.0032 - .013 - .026	.013 - .029 - .045	.0097 - .023 - .036	.0032 - .0097 - .019	0 - .013 - .019
MTORC1	0 - .01 - .031	0 - 0 - 0	0 - .01 - .031	0 - .01 - .021	0 - .021 - .041	0 - .031 - .062	0 - 0 - .01	0 - 0 - 0	0 - 0 - 0	0 - 0 - 0
OPRK1	0 - .083 - .17	0 - 0 - 0	0 - .042 - .12	0 - 0 - 0	0 - .042 - .12	0 - 0 - 0	0 - 0 - 0	0 - 0 - 0	0 - 0 - 0	0 - 0 - 0
PKM2	.0018 - .0055 - .011	.0055 - .015 - .024	0 - .0055 - .011	.0073 - .015 - .022	.0037 - .0092 - .018	.0073 - .015 - .024	.0037 - .011 - .018	.0018 - .0073 - .015	.0018 - .0073 - .015	.0055 - .013 - .022
PPARG	0 - .074 - .15	0 - 0 - 0	0 - .037 - .11	0 - .037 - .11	0 - .037 - .11	0 - 0 - 0	0 - 0 - 0	0 - .074 - .19	0 - 0 - 0	0 - 0 - 0
TP53	0 - .024 - .071	0 - 0 - 0	0 - .024 - .073	0 - .024 - .071	0 - .024 - .071	0 - 0 - 0	0 - .048 - .12	.023 - .071 - .14	0 - 0 - .024	0 - .024 - .048
VDR	.0034 - .0079 - .014	.009 - .015 - .022	.0068 - .012 - .018	.01 - .017 - .024	.0045 - .0091 - .015	.0068 - .012 - .018	.0011 - .0034 - .0068	0034 - .0079 - .012	.015 - .022 - .031	.0034 - .0079 - .012

Difference from Default (Affinity)

-0.2 -0.1 0.0 0.1 0.2

Figure S14: LIT-PCBA NEF1% performance, with 5%–95% confidence intervals, shaded by difference with Default (Affinity). White boxes are not significant (p > 0.0005). Red means the method is better than Default (Affinity) and blue worse.

EF1%

	Default (Affinity)	Default (Pose)	Dense (Affinity)	Dense (Pose)	General (Affinity)	General (Pose)	Vina	Vinardo	RFScore-VS	RFScore-4
ADRB2	0 - 0 - 0	5.9 - 18 - 35	0 - 0 - 0	0 - 12 - 24	0 - 5.9 - 18	0 - 12 - 29	0 - 0 - 0	0 - 0 - 0	0 - 0 - 0	0 - 0 - 0
ALDH1	1.6 - 1.9 - 2.1	.99 - 1.2 - 1.4	1.5 - 1.8 - 2.1	1.2 - 1.4 - 1.6	1.6 - 1.8 - 2.1	.95 - 1.1 - 1.3	1.3 - 1.5 - 1.8	1.2 - 1.4 - 1.6	1.3 - 1.5 - 1.7	.96 - 1.1 - 1.3
ESR1 ago	0 - 15 - 31	0 - 0 - 0	0 - 15 - 31	0 - 7.7 - 23	0 - 7.7 - 23	0 - 0 - 0	0 - 7.7 - 23	0 - 0 - 0	0 - 7.7 - 23	0 - 7.7 - 23
ESR1 ant	2 - 5.9 - 8.9	0 - 3 - 5.9	3 - 7.9 - 11	.99 - 4 - 6.9	.99 - 3 - 5.9	.99 - 3 - 5.9	.99 - 3 - 5.9	0 - .99 - 5	0 - 0 - 2	.99 - 3 - 6.9
FEN1	.81 - 1.9 - 3	1.1 - 2.4 - 3.8	4.1 - 6 - 7.9	7.6 - 10 - 12	.54 - 1.4 - 2.4	.54 - 1.6 - 2.7	1.4 - 2.4 - 3.8	.54 - 1.6 - 2.7	1.6 - 3 - 4.3	.54 - 1.4 - 2.4
GBA	5.4 - 9 - 13	1.8 - 4.2 - 6.6	7.8 - 12 - 16	0 - 1.8 - 3.6	2.4 - 5.4 - 8.4	.6 - 2.4 - 4.8	1.2 - 3.6 - 6	3 - 5.4 - 8.4	0 - 0 - 1.2	1.8 - 3.6 - 6
IDH1	5.1 - 13 - 23	0 - 2.6 - 7.7	2.6 - 7.7 - 15	0 - 2.6 - 7.7	7.7 - 15 - 26	0 - 2.6 - 7.7	0 - 0 - 0	0 - 0 - 0	0 - 7.7 - 15	0 - 2.6 - 7.7
KAT2A	.52 - 1.5 - 3.1	0 - .52 - 1.5	1 - 2.6 - 4.6	0 - 1 - 2.1	0 - 1 - 2.6	0 - .52 - 1.5	0 - .52 - 1.5	0 - 1 - 2.6	1 - 2.6 - 4.6	0 - 1 - 2.6
MAPK1	.65 - 1.6 - 2.6	.65 - 1.6 - 2.9	.32 - 1.3 - 2.6	.97 - 2.3 - 3.9	0 - .97 - 1.9	.32 - 1.3 - 2.6	1.3 - 2.9 - 4.5	.97 - 2.3 - 3.6	.32 - .97 - 1.9	0 - 1.3 - 1.9
MTORC1	0 - 1 - 3.1	0 - 0 - 0	0 - 1 - 3.1	0 - 1 - 2.1	0 - 2.1 - 4.1	0 - 3.1 - 6.2	0 - 0 - 1	0 - 0 - 0	0 - 0 - 0	0 - 0 - 0
OPRK1	0 - 8.3 - 17	0 - 0 - 0	0 - 4.2 - 12	0 - 0 - 0	0 - 4.2 - 12	0 - 0 - 0	0 - 0 - 0	0 - 0 - 0	0 - 0 - 0	0 - 0 - 0
PKM2	.18 - .55 - 1.1	.55 - 1.5 - 2.4	0 - .55 - 1.1	.73 - 1.5 - 2.2	.37 - .92 - 1.8	.73 - 1.5 - 2.4	.37 - 1.1 - 1.8	.18 - .73 - 1.5	.18 - .73 - 1.5	.55 - 1.3 - 2.2
PPARG	0 - 7.5 - 15	0 - 0 - 0	0 - 3.7 - 11	0 - 3.7 - 11	0 - 3.7 - 11	0 - 0 - 0	0 - 0 - 0	0 - 7.5 - 19	0 - 0 - 0	0 - 0 - 0
TP53	0 - 1.3 - 3.8	0 - 0 - 0	0 - 1.3 - 3.9	0 - 1.3 - 3.8	0 - 1.3 - 3.8	0 - 0 - 0	0 - 2.6 - 6.4	1.2 - 3.8 - 7.7	0 - 0 - 1.3	0 - 1.3 - 2.6
VDR	.34 - .79 - 1.4	.9 - 1.5 - 2.2	.68 - 1.2 - 1.8	1 - 1.7 - 2.4	.45 - .91 - 1.5	.68 - 1.2 - 1.8	.11 - .34 - .68	.34 - .79 - 1.2	1.5 - 2.2 - 3.1	.34 - .79 - 1.2

Difference from Default (Affinity)

-20 -10 0 10 20

Figure S15: LIT-PCBA EF1% performance, with 5%–95% confidence intervals, shaded by difference with Default (Affinity). White boxes are not significant (p > 0.0005). Red means the method is better than Default (Affinity) and blue worse.

AUC

	Default (Affinity)	Default (Pose)	Dense (Affinity)	Dense (Pose)	General (Affinity)	General (Pose)	Vina	Vinardo	RFScore-VS	RFScore-4
ADRB2	.4 - .52 - .62	.47 - .6 - .73	.41 - .51 - .61	.39 - .53 - .66	.46 - .57 - .68	.54 - .65 - .76	.26 - .37 - .49	.33 - .43 - .53	.54 - .63 - .73	.28 - .38 - .49
ALDH1	.59 - .6 - .6	.48 - .48 - .49	.6 - .6 - .61	.48 - .49 - .5	.57 - .58 - .58	.47 - .48 - .49	.58 - .58 - .59	.56 - .56 - .57	.49 - .5 - .5	.57 - .58 - .58
ESR1 ago	.6 - .71 - .83	.31 - .45 - .59	.6 - .72 - .82	.35 - .51 - .67	.55 - .69 - .82	.25 - .38 - .52	.57 - .7 - .83	.6 - .73 - .85	.38 - .54 - .69	.53 - .67 - .79
ESR1 ant	.68 - .72 - .76	.45 - .5 - .55	.66 - .7 - .75	.47 - .52 - .56	.65 - .69 - .72	.44 - .49 - .54	.6 - .65 - .7	.63 - .68 - .72	.37 - .41 - .46	.66 - .7 - .74
FEN1	.53 - .55 - .58	.57 - .6 - .62	.59 - .62 - .64	.6 - .63 - .66	.48 - .51 - .53	.55 - .58 - .6	.54 - .56 - .59	.55 - .58 - .6	.6 - .62 - .65	.46 - .48 - .51
GBA	.68 - .71 - .75	.49 - .53 - .57	.69 - .72 - .75	.47 - .51 - .54	.68 - .71 - .74	.53 - .56 - .6	.64 - .68 - .71	.67 - .71 - .74	.58 - .62 - .66	.6 - .64 - .68
IDH1	.61 - .69 - .77	.37 - .44 - .51	.65 - .71 - .78	.43 - .51 - .59	.57 - .66 - .74	.35 - .42 - .48	.51 - .58 - .64	.46 - .54 - .62	.47 - .56 - .64	.54 - .6 - .67
KAT2A	.37 - .41 - .45	.48 - .51 - .54	.39 - .43 - .47	.48 - .51 - .55	.38 - .42 - .45	.46 - .49 - .53	.4 - .43 - .48	.38 - .41 - .45	.51 - .54 - .58	.38 - .42 - .45
MAPK1	.6 - .62 - .64	.49 - .52 - .55	.62 - .64 - .66	.5 - .53 - .56	.55 - .57 - .59	.47 - .5 - .53	.62 - .65 - .67	.62 - .64 - .67	.5 - .53 - .55	.58 - .6 - .63
MTORC1	.44 - .49 - .53	.44 - .49 - .54	.44 - .48 - .53	.46 - .51 - .56	.44 - .49 - .53	.51 - .55 - .6	.39 - .44 - .48	.43 - .48 - .52	.47 - .52 - .57	.38 - .43 - .48
OPRK1	.74 - .8 - .87	.4 - .47 - .54	.67 - .75 - .83	.33 - .41 - .5	.71 - .79 - .85	.39 - .47 - .55	.48 - .55 - .62	.46 - .53 - .61	.65 - .72 - .79	.65 - .71 - .78
PKM2	.6 - .61 - .63	.51 - .53 - .55	.58 - .6 - .62	.5 - .52 - .54	.6 - .62 - .64	.49 - .51 - .53	.58 - .6 - .62	.6 - .61 - .63	.44 - .46 - .49	.6 - .62 - .63
PPARG	.7 - .76 - .83	.54 - .62 - .69	.65 - .73 - .79	.57 - .65 - .73	.69 - .75 - .81	.5 - .58 - .65	.65 - .72 - .79	.68 - .76 - .83	.4 - .5 - .59	.69 - .75 - .81
TP53	.52 - .56 - .6	.42 - .47 - .52	.54 - .6 - .64	.44 - .49 - .54	.47 - .52 - .57	.43 - .48 - .53	.57 - .62 - .67	.56 - .61 - .66	.48 - .53 - .59	.56 - .6 - .64
VDR	.37 - .39 - .4	.54 - .56 - .57	.43 - .45 - .47	.57 - .59 - .6	.34 - .36 - .37	.48 - .5 - .51	.4 - .41 - .43	.43 - .44 - .46	.57 - .59 - .6	.37 - .38 - .4

Difference from Default (Affinity)

-0.2 -0.1 0.0 0.1 0.2

Figure S16: LIT-PCBA AUC performance, with 5%–95% confidence intervals, shaded by difference with Default (Affinity). White boxes are not significant (p > 0.0005). Red means the method is better than Default (Affinity) and blue worse.

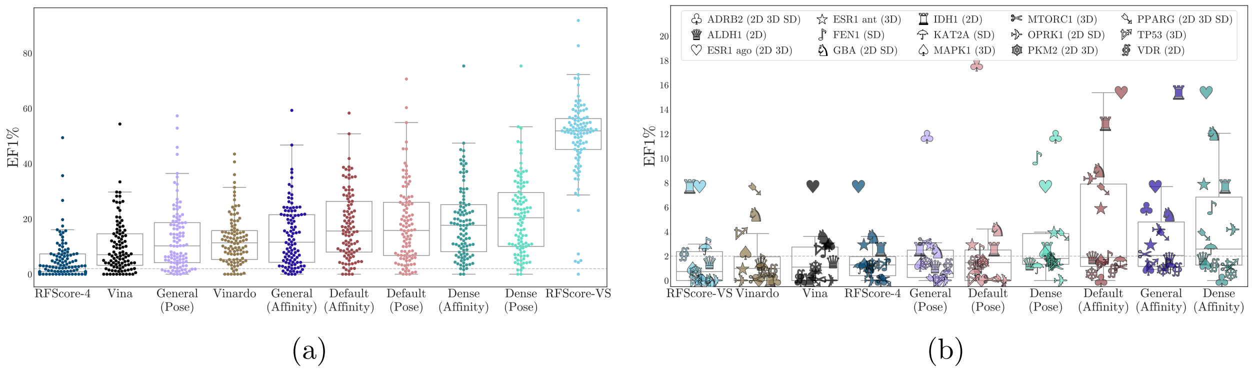

Figure S17: Assessment of virtual screening performance using EF1% on (a) DUD-E and (b) LIT-PCBA. The x-axis is sorted in order of increasing median performance. Each data point is the performance of the method on a single target. Note the different y-axis scales: DUD-E targets demonstrate significantly better enrichment. LIT-PCBA targets are assigned a unique symbol; the legend notes which method achieved an EF1%> 2 in **?**, where ‘1D’ signifies fingerprints, ‘2D’ signifies ligand shape shape, and ‘3D’ signifies docking with Surflex-Dock. Note that while EF1% = 1 is equivalent to random performance, we have annotated EF1% = 2 since that was the minimum enrichment required for inclusion in LIT-PCBA.[?]

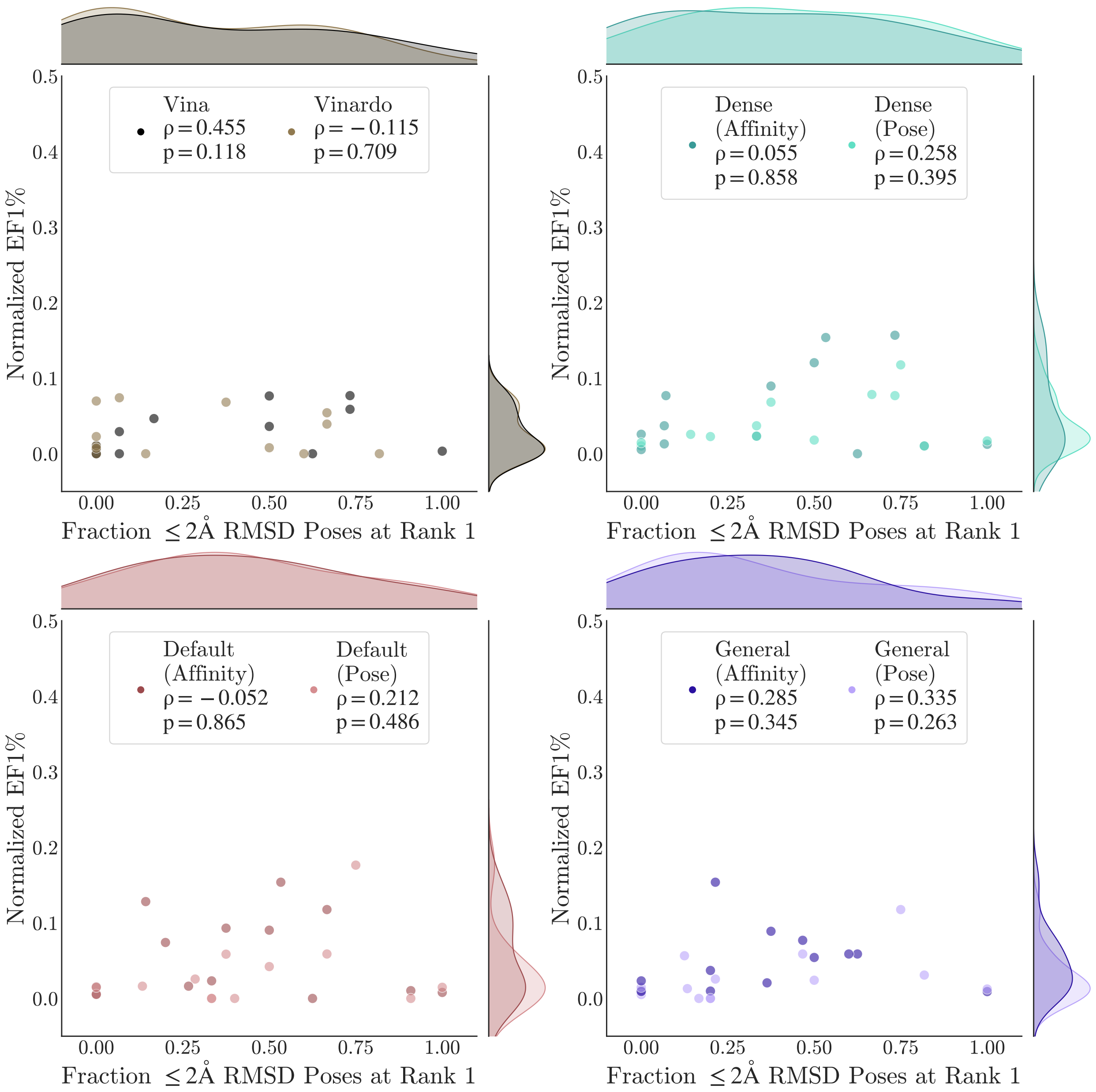

Figure S18: Correlation between normalized EF1% for the virtual screening task and the fraction of compounds with a good pose ranked first in the pose prediction task. The Spearman correlation and its p-value is shown (there are no statistically significant correlations).

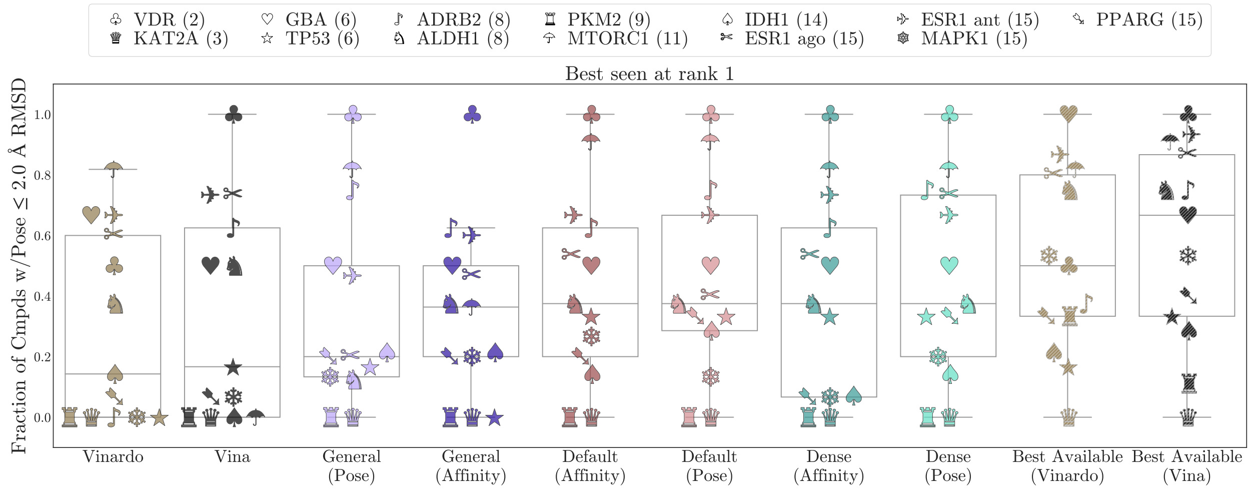

Figure S19: Boxplots showing the fraction of compounds, per-target, that have a pose $\leq$ 2Å RMSD from the provided crystal pose ranked first by each method when cross-docked to non-cognate structures. The two plots with hatched markers show the fraction of compounds for which such a pose was *sampled*. Numbers in the legend indicate the number of reference receptors provided for each target.

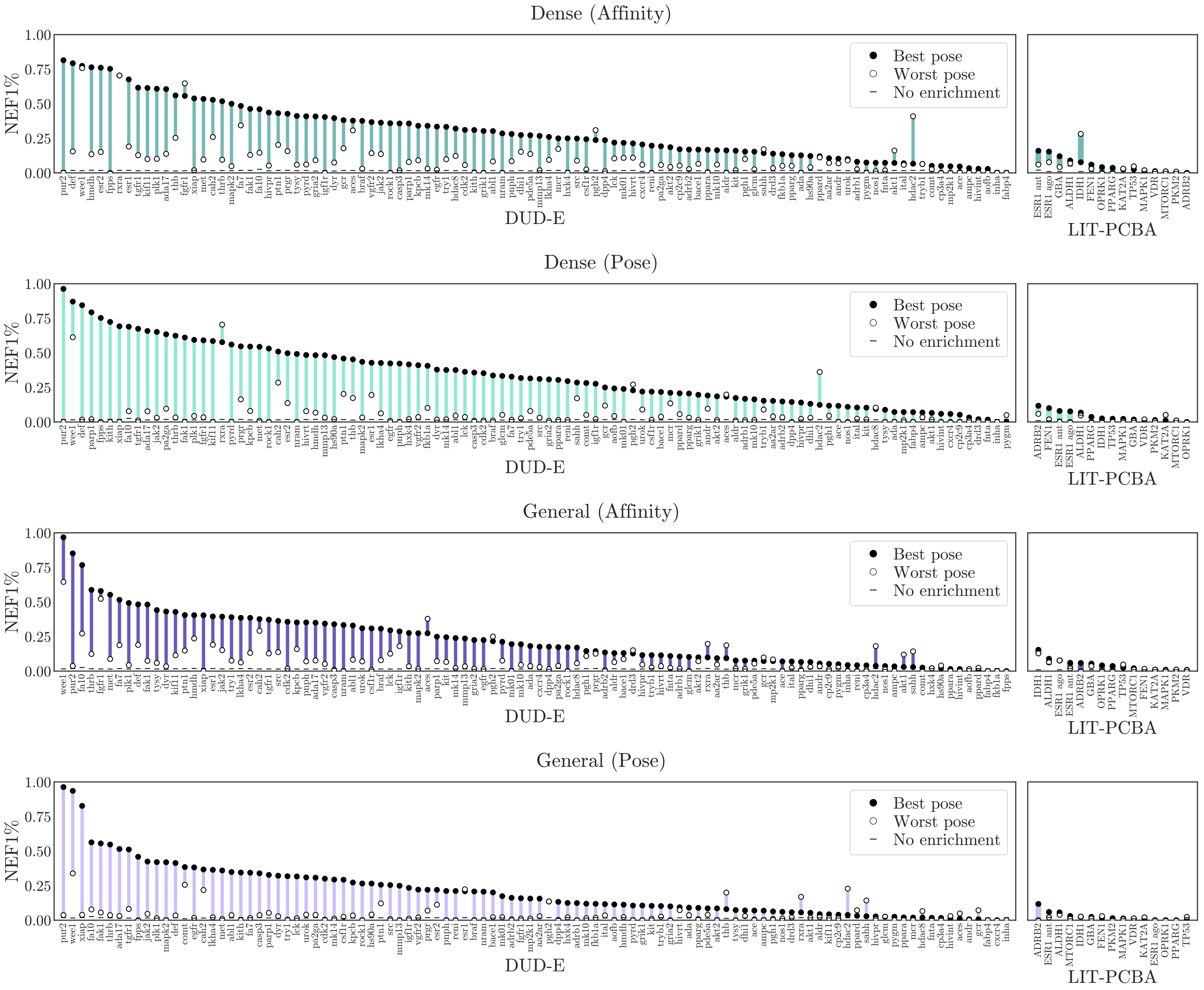

Figure S20: Pose sensitivity assessment of CNN models. For each target the difference in NEF1% when basing the score on the top-ranked versus the bottom-ranked pose is shown.

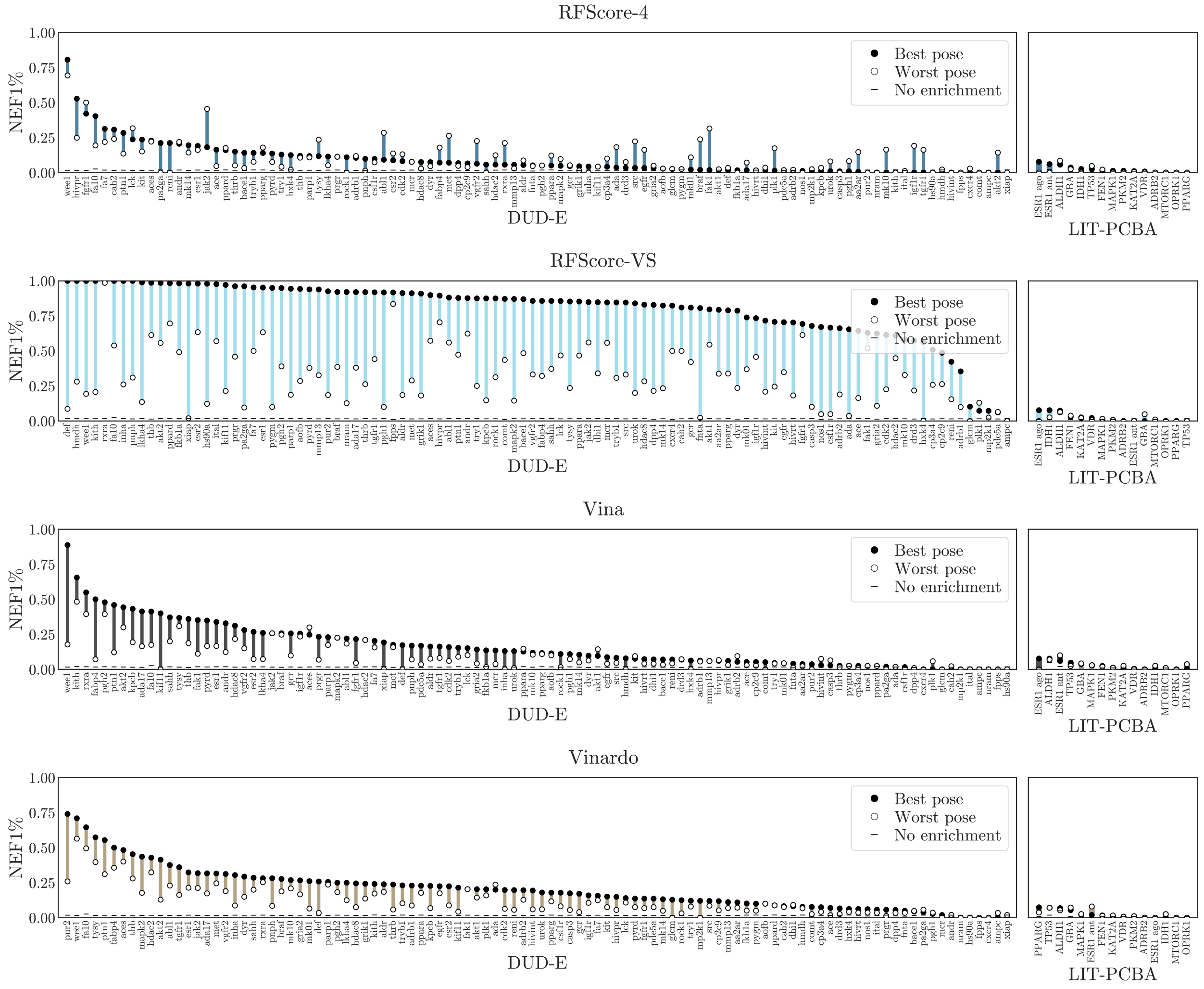

Figure S21: Pose sensitivity assessment of non CNN models. For each target the difference in NEF1% when basing the score on the top-ranked versus the bottom-ranked pose is shown. Note that RFScore-VS was trained on the DUD-E.

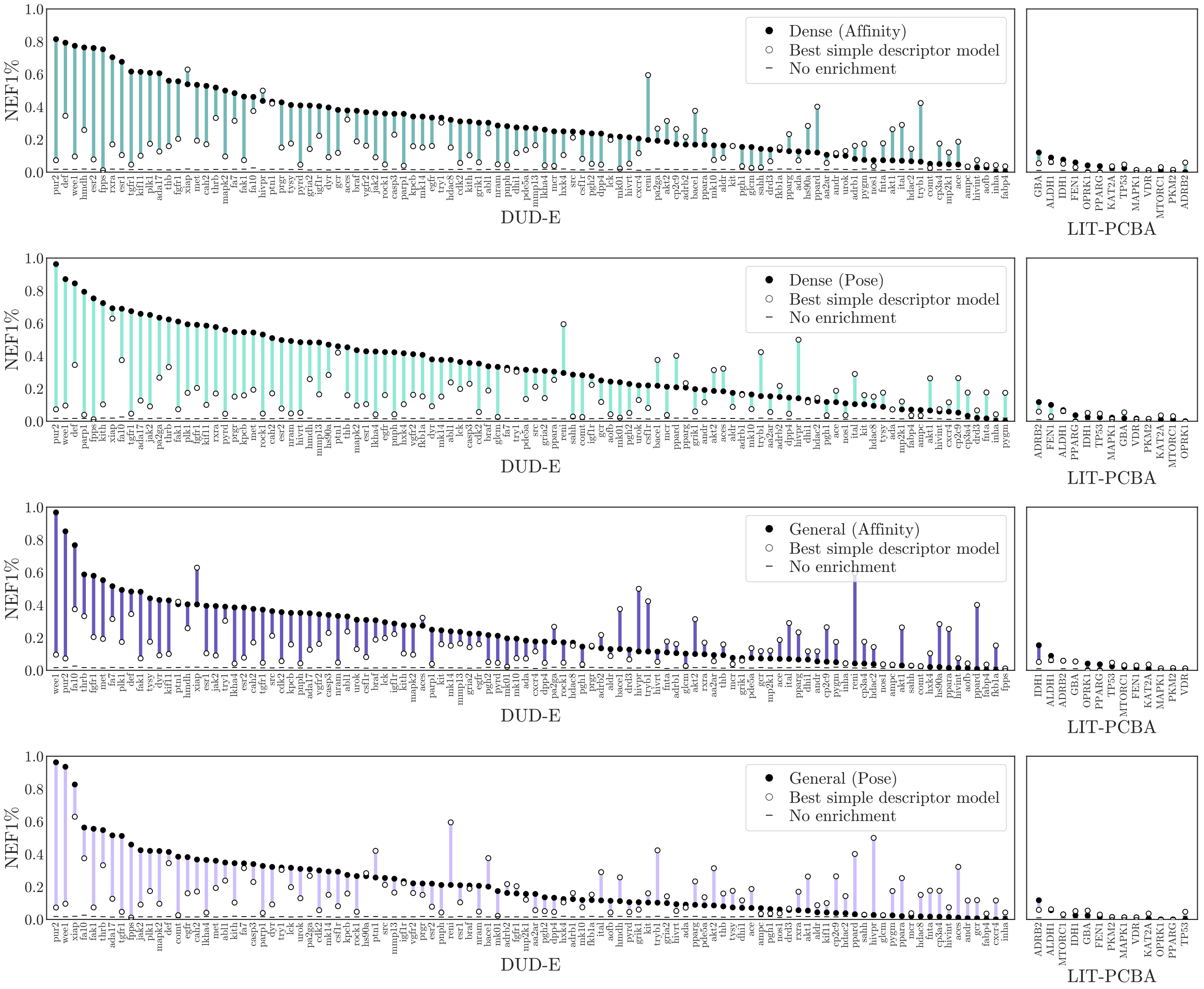

Figure S22: Comparison of CNN models to simple descriptor models. The best achievable performance of a model trained on simple molecular descriptors to predict binding affinities is shown. The x-axes are sorted independently by the NEF1% of the best scoring pose.

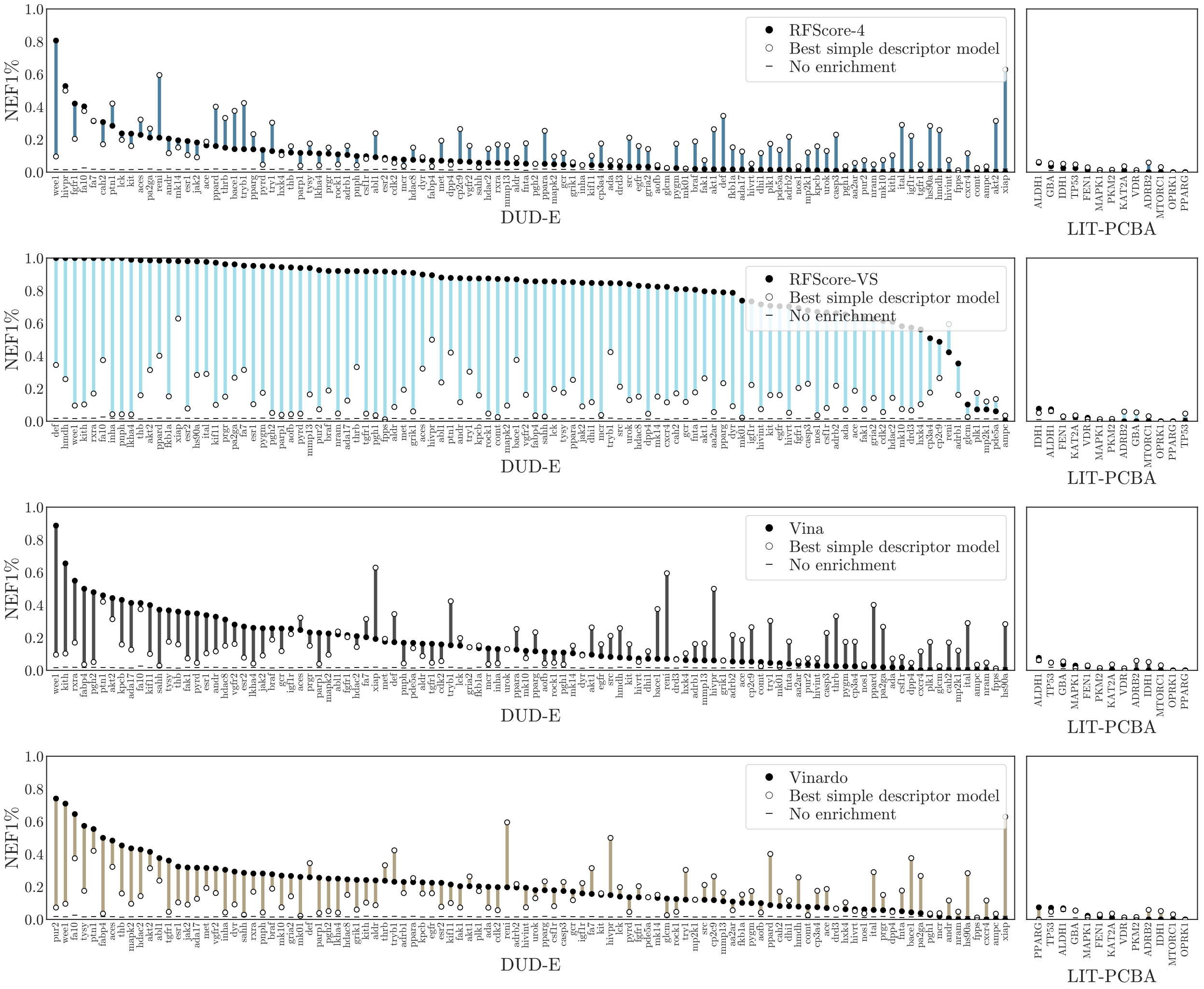

Figure S23: Comparison of CNN models to simple descriptor models. The best achievable performance of a model trained on simple molecular descriptors to predict binding affinities is shown. The x-axes are sorted independently by the NEF1% of the best scoring pose.

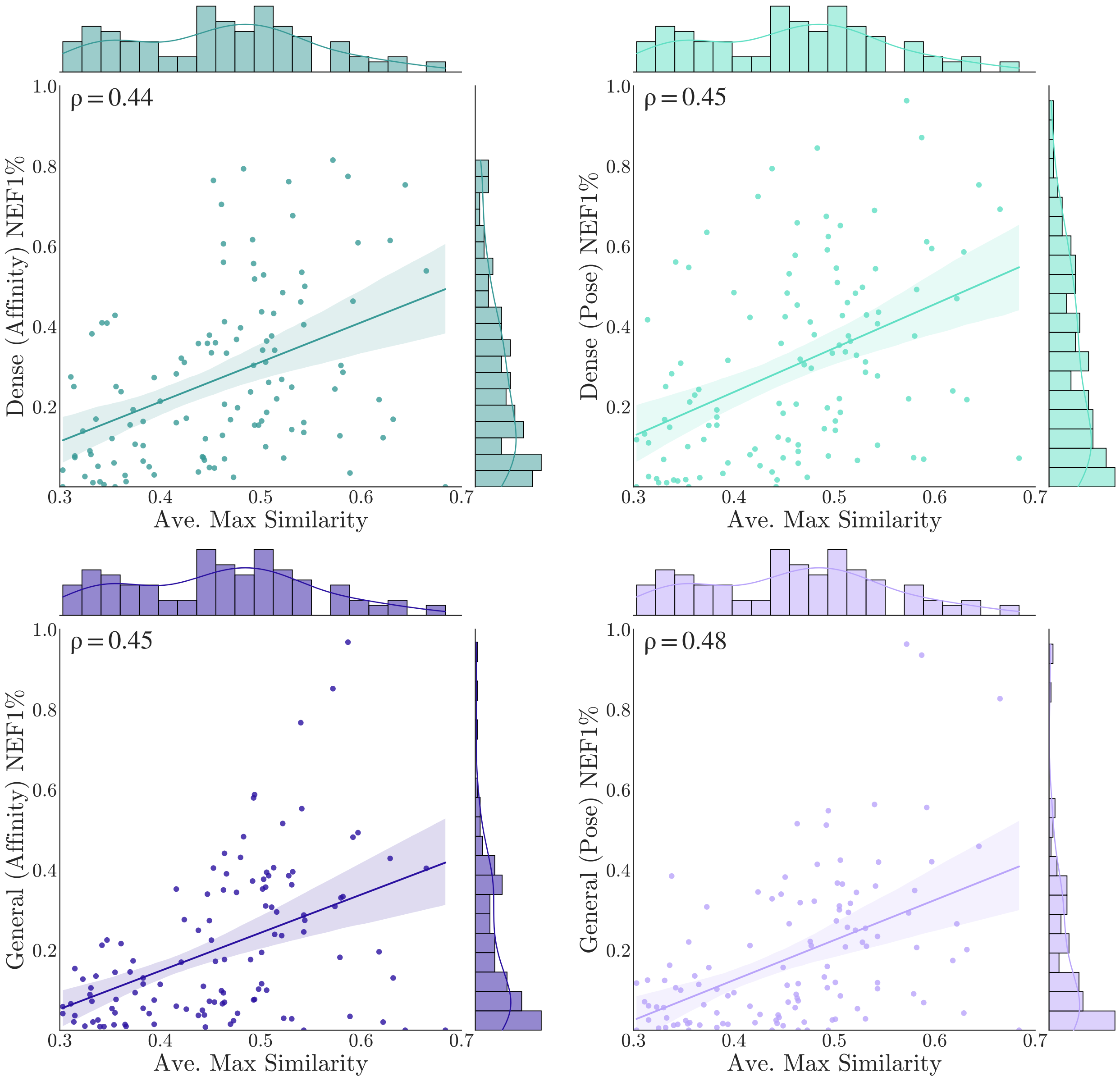

Figure S24: Correlation between similarity with training set and early enrichment performance for the CNN models. For each benchmark, the average of the maximum similarity between active compounds and the PDBbind General set is computed using the Tanimoto coefficient of ECFP4 fingerprints. Only actives are considered since the training set does not include any inactive compounds.

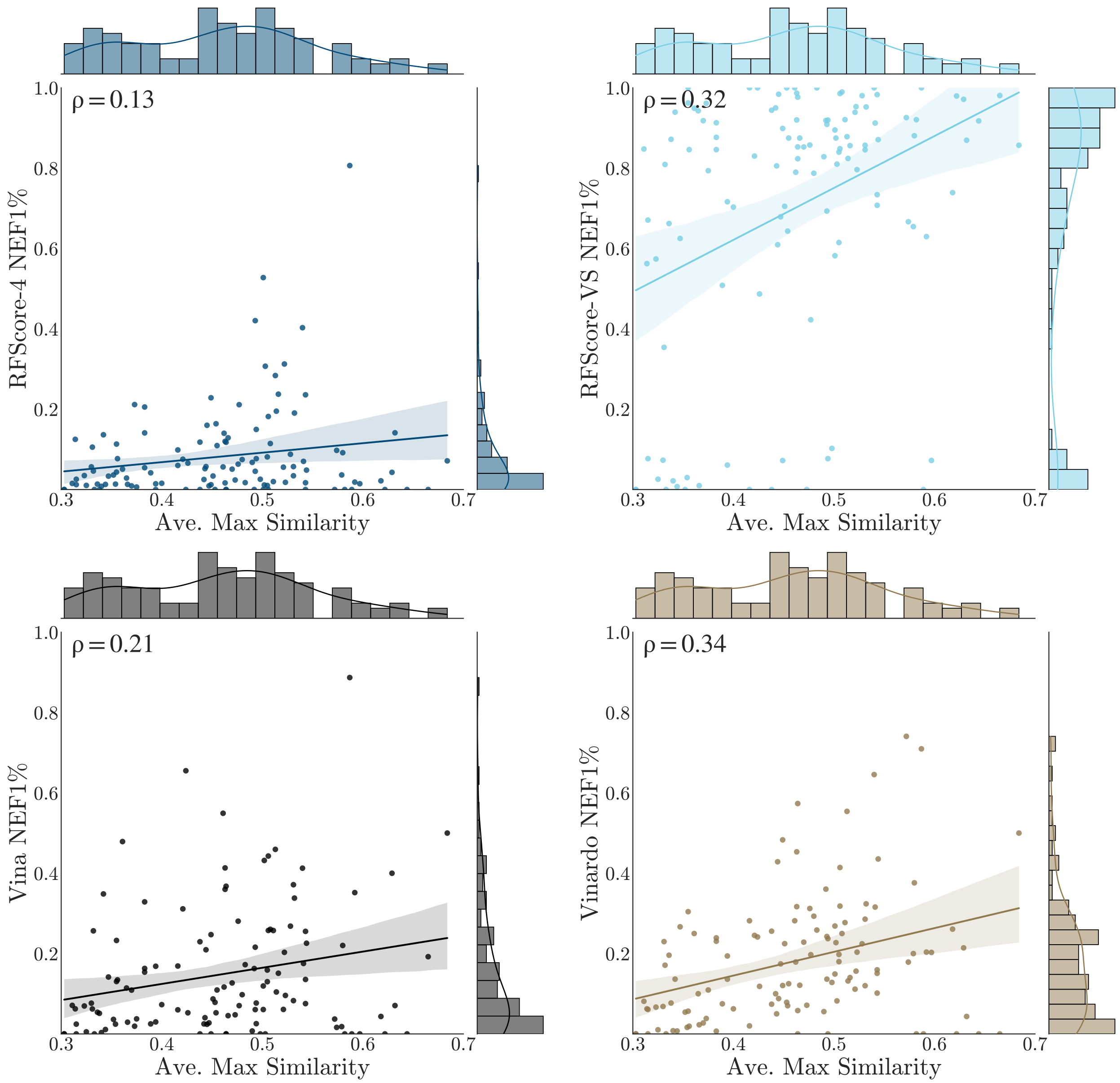

Figure S25: Correlation between similarity with training set and early enrichment performance for the non-CNN models. For each benchmark, the average of the maximum similarity between active compounds and the PDBbind General set is computed using the Tanimoto coefficient of ECFP4 fingerprints. Only actives are considered since the training set does not include any inactive compounds. Note that RFScore-VS used DUD-E for training.

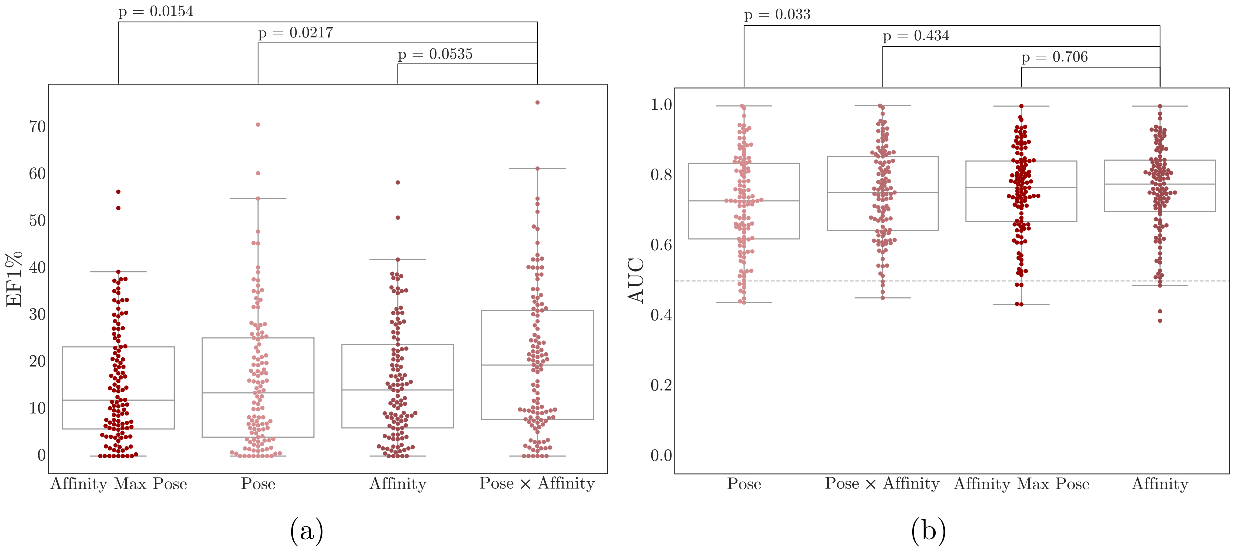

Figure S26: Performance of pose/affinity score combinations measured with (a) EF1% and (b) AUC. Pose and affinity scores are combined either by taking the predicted affinity of the pose with the best pose score, or by multiplying the affinity and pose scores. Performance of the Default ensemble on both DUD-E and LIT-PCBA targets is shown. P-values are computed using the two-sided Mann-Whitney U rank test. The x-axes are independently sorted by the median value.

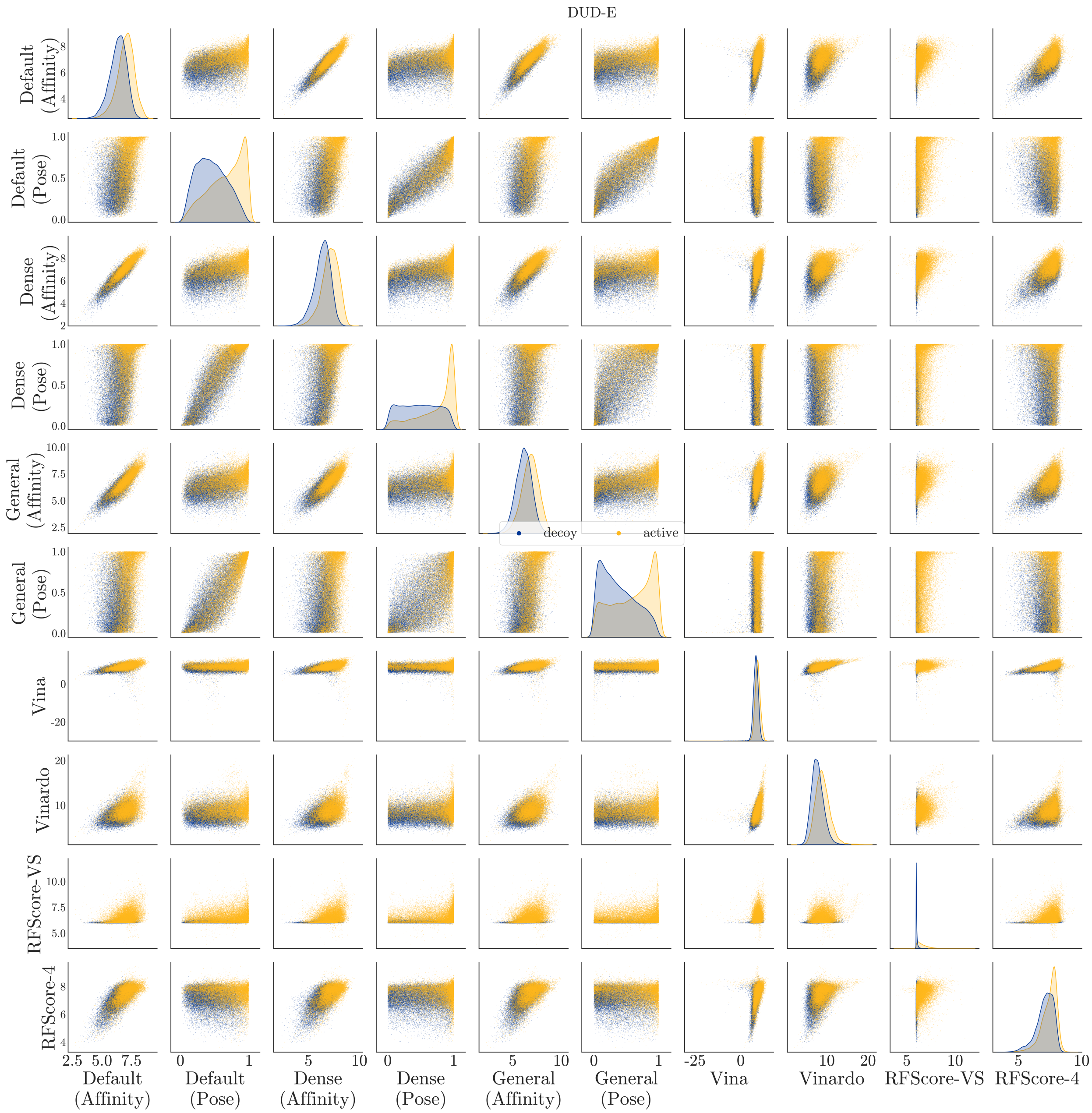

Figure S27: Score distributions and correlations for the different methods tested for DUD-E, separated by class. Note that RFScore-VS was trained on DUD-E. For visualization purposes, decoys were randomly downsampled to the same size as the actives.

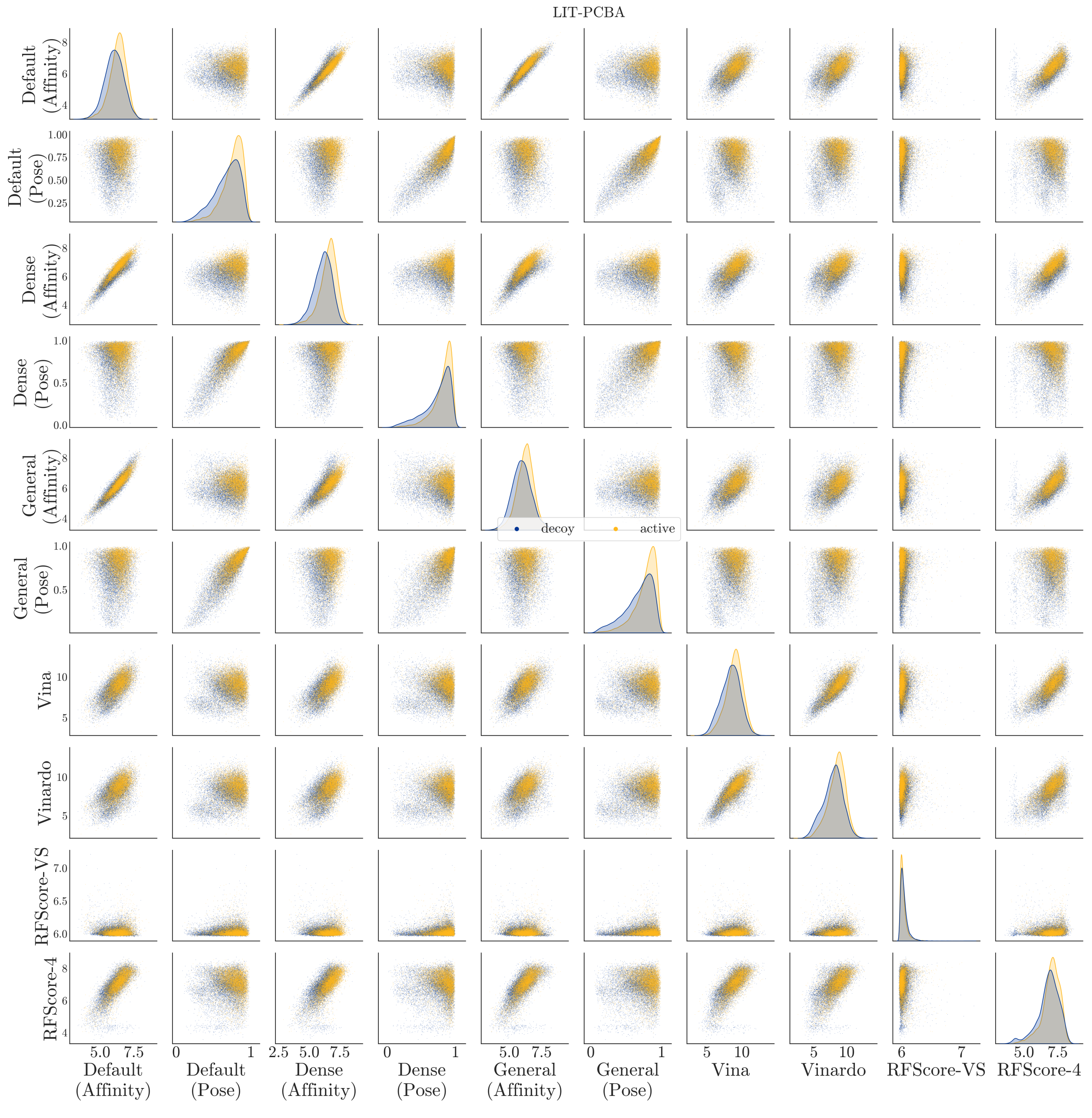

Figure S28: Score distributions and correlations for the different methods tested for LIT-PCBA, separated by class. For visualization purposes, decoys were randomly downsampled to the same size as the actives.

References

(1) Adeshina, Y.; Deeds, E.; Karanicolas, J. Machine learning classification can reduce false positives in structure-based virtual screening. *bioRxiv* **2020**,

(2) Dietterich, T. G. Approximate statistical tests for comparing supervised classification learning algorithms. *Neural computation* **1998**, *10*, 1895–1923.

(3) Nicholls, A. Confidence limits, error bars and method comparison in molecular modeling. Part 1: the calculation of confidence intervals. *Journal of computer-aided molecular design* **2014**, *28*, 887–918.

(4) Zhao, W.; Hevener, K. E.; White, S. W.; Lee, R. E.; Boyett, J. M. A statistical framework to evaluate virtual screening. *BMC bioinformatics* **2009**, *10*, 225.

(5) Francoeur, P. G.; Masuda, T.; Sunseri, J.; Jia, A.; Iovanisci, R. B.; Snyder, I.; Koes, D. R. Three-dimensional convolutional neural networks and a cross-docked data set for structure-based drug design. *Journal of Chemical Information and Modeling* **2020**, *60*, 4200–4215.
